# Testing for UIP-Type Relationships: Nonlinearities, Monetary Announcements and Interest Rate Expectations

**DOI:** 10.1007/s11079-021-09640-8

**Published:** 2022-02-14

**Authors:** Christina Anderl, Guglielmo Maria Caporale

**Affiliations:** 1 London South Bank University, London, UK; 2 Department of Economics and Finance, Brunel University London, London, UB8 3PH UK

**Keywords:** UIP, Exchange rate, Nonlinearities, Asymmetric adjustment, CVAR (Cointegrated VAR), STCVAR (Smooth Transition Cointegrated VAR), Interest rate expectations, Interest rate announcements, C32, F31, G15

## Abstract

This paper tests for UIP-type relationships by estimating first a benchmark linear Cointegrated VAR including the nominal exchange rate and the interest rate differential as well as central bank announcements, and then a Smooth Transition Cointegrated VAR (STCVAR) model incorporating nonlinearities and also taking into account the role of interest rate expectations. The analysis is conducted for five inflation targeting countries (the UK, Canada, Australia, New Zealand and Sweden) and three non-targeters (the US, the Euro-Area and Switzerland) using daily data from January 2000 to December 2020. While we cannot confirm the validity of UIP in its strictest theoretical sense, we find evidence for the existence of an equilibrium relationship between the exchange rate and the interest rate differential. Specifically, the nonlinear framework appears to be more appropriate to capture the adjustment towards the long-run equilibrium, since the estimated speed of adjustment is substantially faster and the short-run dynamic linkages more significant. Further, interest rate expectations play an important role: a fast adjustment only occurs when the market expects the interest rate to increase in the near future, namely central banks are perceived as more credible when sticking to their goal of keeping inflation at a low and stable rate. Also, central bank announcements have a more sizeable short-run effect in the nonlinear model. Finally, the equilibrium relationship between the exchange rate and the interest rate differential holds better in inflation targeting countries, where monetary authorities appear to achieve a higher degree of credibility.

## Introduction

Uncovered Interest Rate Parity (UIP) is one of the central tenets of international finance; it links interest rate differentials to expected exchange rate changes, and, in particular, it predicts that high yield currencies should depreciate. Various methods have been used to test it empirically; these include simple Ordinary Least Squares (OLS) for estimating the slope coefficient in the UIP relation in early studies (see, for instance, Froot and Thaler 1990; Engel 1996), equilibrium-correction models of the term structure (Clarida and Taylor 1997) and cointegration tests between UIP and PPP to account for the interaction of goods and capital markets (Johansen and Juselius 1992). Most empirical papers reject the validity of UIP in the short run (see Engel 1996; Sarno, 2005; Banerjee and Singh 2006), and in some cases even in the long run (Lothian 2016). These findings represent a puzzle for which a range of explanations have been offered, such as the existence of a time-varying risk premium (Li et al. 2012; Jiang et al. 2013), the occurrence of rational bubbles (Obstfeld 1987; Canterbery 2000), or deviations from rationality of market participants (Gregory 1987; Chinn and Quayyum 2012).

While there is plenty of evidence on whether or not UIP holds under various monetary policy regimes (Lacerda et al. 2010), in the case of inflation targeting the existing studies only consider emerging markets (Coulibaly and Kempf 2019). The present paper instead examines this issue using daily data for five inflation targeting developed countries, namely the UK, Canada, Australia, New Zealand and Sweden, over the period from January 2000 to December 2020; for comparison purposes, the analysis is also carried out for three economies with alternative monetary regimes, namely the US, the Euro-Area and Switzerland (Neumann and Von Hagen 2002). This classification is based on how central banks identify themselves, despite the fact that they might in fact have adopted different monetary approaches at different points in time. As a first step, a linear Cointegrated Vector Autoregressive (CVAR) benchmark model (Juselius 2018) is estimated which yields a UIP-type relationship between the exchange rate and the interest rate differential; its specification also takes into account the effects of central bank announcements of interest rate changes on the exchange rate and the interest rate differential. However, the more recent literature suggests the possible presence of nonlinearities in the UIP relation; for instance, Smooth Transition Regression models have been found to outperform linear ones in explaining the UIP puzzle (Sarno et al. 2006; Li et al. 2013). Therefore a Smooth Transition Cointegrated Vector Autoregressive (STCVAR) model (Ripatti 2001) is also estimated; the transition variable is interest rate expectations, which is an indicator of central bank credibility often neglected in empirical studies on inflation targeting despite its possible importance; specifically, we use the change in the 30-day interest rate, which is shown to be preferable to alternative transition variables.

The layout of this paper is as follows. Section 2 briefly reviews the relevant literature; Sect. 3 outlines the methodology; Sect. 4 presents the data and discusses the empirical results; Sect. 5 offers some concluding remarks.

## Literature Review

The validity of UIP has been investigated in numerous papers and from various angles. Estimation methods range from simple linear regressions (Lothian and Wu 2011; Moore and Roche 2010) to more complex multivariate nonlinear models (Sarno et al. 2005). As for cointegration tests, several studies have carried them out to analyse the linkages between spot and forward exchange rates and have provided mixed evidence for the empirical validity of CIP (Covered Interest Rate Parity– e.g., Brenner and Kroner 1995; Zivot, 2000; Clarida et al. 2003); by contrast, there exists only a relatively small set of papers assessing UIP by testing for cointegration between exchange rates and interest rate differentials – again the results have been mixed (Georgoutsos and Kouretas 2002; Weber 2006).

The more recent literature emphasises the importance of taking into account nonlinearities when investigating the UIP relation. For instance, Lyons (2001) estimated a nonlinear model in which deviations of UIP are highly persistent; this finding is explained by the lower Sharpe ratio of the forward rates, which move trade to more lucrative investment opportunities. Sarno et al. (2005) suggested that the forward bias commonly observed in the empirical literature might be a less suitable explanation of forward market inefficiencies than previously assumed. They applied a Smooth Transition Regression (STR) model and found evidence of significant nonlinearities in the UIP relation; in particular, asymmetric deviations from UIP are found to be small, but more persistent, the closer they are to the UIP equilibrium. Baillie and Kilic (2006) analysed nonlinearities in the context of a Logistic Smooth Transition Regression (LSTR) of the spot exchange rate and the lagged forward premium model with different transition variables; their results imply a nonlinear relation, with forward premium being the transition variable. Sarno et al. (2006) estimated a Smooth Transition Regression model for UIP with the expected excess return as the transition variable and also found that deviations from UIP exhibit significant nonlinearities. Using a LSTR model with the risk-adjusted forward premium as the transition variable, Amri (2008) found evidence of nonlinearities in the relation between expected exchange rate changes and the lagged forward premium. Applying the same methodology, but with different transition variables related to currency trading strategies, Baillie and Chang (2011) showed that UIP holds only when carry trade strategies are perceived as profitable—when they are not, their results confirm those of Lyons (2001) and provide additional support for nonlinearities in the UIP relation. Li et al. (2013) followed a similar approach and found empirical support for UIP when using exchange rate volatility and the Sharpe ratio as transition variables, but not when using instead the interest rate differential.

Another issue investigated more recently in the context of UIP is the role of interest rate expectations, which had been found previously to affect the slope of the yield curve (Cook and Hahn 1990), financial ratios (Chen and Ainina 1994) and the exchange rate (Mauleón 1998). Juselius and Stillwagon (2018) reported that interest rate forecasts are the primary source of deviations from the exchange rate and interest rate equilibrium and found an important role for speculative bubbles. Several studies provide evidence that central bank announcements strongly influence interest rate expectations (Moniz and De Jong 2014), and that some central banks even use the content of their announcements intentionally to influence interest rate expectations (Tietz 2019). Announcements containing policy rate decisions have been found to have a particularly strong effect on asset prices, including the exchange rate and interest rate, compared to other types of announcements (Sager and Taylor 2004; Rosa and Verga 2018).

## Empirical Methodology

To investigate the issues of interest, as a first step we estimate a linear Cointegrated VAR as in Juselius (2018) for the UIP relation. In order for two variables to cointegrate and be linked by a long-run equilibrium relationship, they must exhibit the same non-zero order of integration. Therefore, to establish if this condition is satisfied, prior to this estimation we perform unit root tests for the individual series, specifically the Dickey Fuller Generalised Least Squares (DF-GLS) and the Kwiatkowski–Phillips–Schmidt–Shin (KPSS) tests. We the employ the Johansen (1991) cointegration trace and eigenvalue tests to test for cointegration between the series.

### The Cointegrated VAR (CVAR) Model

The standard linear Cointegrated VAR model takes the following general form:

1 $$\begin{array}{c}\Delta x_t=\Pi x_{t-1}+\sum\limits_{i=1}^{q}{\gamma }\Delta x_{t-i}+\Phi D_t+\varepsilon_t\end{array}\\$$

where ${x}_{t}$ is a vector with the series under examination, Δ is the difference operator, $\Pi =\theta {\beta }^{^{\prime}}$ is a matrix given by the product of two vectors including the adjustment and the cointegrating coefficients respectively, ${\Gamma }_{i}$ is the coefficient matrix of the parameters governing the short-run behaviour of the variables, ${D}_{t}$ is a vector of exogenous dummy variables and $\Phi$ the corresponding coefficient matrix. The model has r cointegrating relations and p endogenous variables; if cointegration holds, whenever the endogenous variables are pushed away from the long-run equilibrium by exogenous shocks, they gradually revert to it, the size of the coefficient $\theta$ representing the adjustment speed. Our empirical CVAR model is specified as follows:

$$\Delta {s}_{t}=\theta {\beta }^{{^{\prime}}}{x}_{t-1}+\sum_{i=1}^{q}{\gamma }_{11,i}\Delta {s}_{t-i}+\sum_{i=1}^{q}{\gamma }_{12,i}\Delta {\tilde{i }}_{t-i}+{\varphi }_{1}{d}_{p}+{\varphi }_{2}{d}_{n}+{\varepsilon }_{t}$$

2 $$\begin{array}{c}\Delta {\tilde{i }}_{t}=\theta {\beta }^{{^{\prime}}}{x}_{t-1}+\sum\limits_{i=1}^{q}{\gamma }_{21,i}\Delta {s}_{t-i}+\sum\limits_{i=1}^{q}{\gamma }_{22,i}\Delta {\tilde{i }}_{t-i}+{\varphi }_{1}{d}_{p}+{\varphi }_{2}{d}_{n}+{\varepsilon }_{t}\end{array}$$

where ${s}_{t}$ is the nominal exchange rate, ${\tilde{i }}_{t}={i}_{t}-{i}_{t}^{*}$ is the difference between the domestic and foreign interest rates, and ${d}_{p}$ and ${d}_{n}$ are two dummies corresponding respectively to the announcement dates for interest rate increases and decreases – they are set equal to 1 on the announcement date and 0 elsewhere and only enter the short-run deterministic component of the model, thus capturing the transitory impulse effects of policy changes without affecting the long-run UIP mechanism (Juselius 2018). All other variables are defined as before. The lag length is chosen using appropriate lag selection criteria, such as the Likelihood-Ratio (LR) Test, to select the most parsimonious specification which ensures that there is no serial correlation.

When estimating a CVAR model, there are two important issues to consider to specify it correctly. One is whether or not deterministic trends should be included in the short-run dynamics and/or the long-run equilibrium relations. In our case they are not required, since the exchange rate and interest rates tend to have a zero mean growth rate and any trending behaviour should be stochastic. However, Juselius (2017) suggests to test for the order of integration of the variables within the system. For this purpose we employ a test of the hypothesis that the individual variables are at most I(1) versus being closer to I(2) after the model has been specified. The other issue is that model identification is only possible when appropriate restrictions are placed on the model parameters. Long-run restrictions have to satisfy the identification rank conditions for the model (Juselius 2018). In order for economic identification of the short-run structure to be possible, the residuals need to be uncorrelated. Therefore, we perform the Breusch-Godfrey Lagrange Multiplier (LM) test for residual serial correlation. In addition, we test the CVAR models for their stability.

### The Smooth Transition Cointegrated VAR Model

Linear models might be unsuitable for the UIP relation as the error correction coefficient might change in a nonlinear fashion. Therefore, a nonlinear Smooth Transition Cointegrated Vector Autoregressive (STCVAR) model (Ripatti 2001), which allows for asymmetric dynamic adjustment to the UIP equilibrium relationship, might be more appropriate. The general model takes the following form:

3 $$\begin{array}{c}\Delta {x}_{t}=\theta {\beta }^{{^{\prime}}}{x}_{t-1}\cdot G\left({z}_{t}\right)+\left(\sum\limits_{i=1}^{q}{\Gamma }_{i}\Delta {x}_{t-i}\right)\cdot G\left({z}_{t}\right)+{\Phi D}_{t}\cdot G\left({z}_{t}\right)+{\varepsilon }_{t}\end{array}$$

where ${x}_{t}$ is the $\left(p\times 1\right)$ vector of the series of interest, Δ is again the difference operator, ${\Gamma }_{i}$ is the $\left(p\times p\right)$ matrix of the short-run coefficients, ${D}_{t}$ is a vector of dummy variables with a parameter matrix $\Phi$, and as before the $\theta$ and *β* vectors include the adjustment speed and cointegrating coefficients respectively. $G\left({z}_{t}\right)=diag\left\{{G}_{1}\left({\gamma }_{1},{c}_{1},{z}_{t}\right),\dots ,{G}_{m}\left({\gamma }_{m},{c}_{m},{z}_{t}\right)\right\}$ is the transition function, where $\gamma$ is the slope parameter, $c$ is the transition value and ${z}_{t}$ is the transition variable. The transition function allows the parameters of the model to change smoothly from one regime to the next as a function of the transition variable ${z}_{t}$.

The transition variable in our empirical specification is the change in the 30-day interest rate, which can be seen as an indicator of changes in interest rate expectations. Central bank meetings and decisions about changes in the interest rate generally occur in monthly cycles. Therefore the 30-day interest rate should not vary greatly over the span of a month if interest rate expectations are aligned with the official monetary policy rate (Connolly and Kohler 2004). If instead it does, this implies a change in market expectations of the monetary policy rate in the near future and can indicate that the central bank is not perceived as fully credible. The empirical model corresponding to Eq. (3) can be written as follows:

$$\begin{array}{c}\Delta {s}_{t}=\theta {\beta }^{{^{\prime}}}{x}_{t-1}\cdot G\left({z}_{t}\right)+\left(\sum\limits_{i=1}^{q}{\gamma }_{11,i}\Delta {s}_{t-i}+\sum\limits_{i=1}^{q}{\gamma }_{12,i}\Delta {\tilde{i }}_{t-i}\right)\cdot G\left({z}_{t}\right)+\left({\varphi }_{1}{d}_{p}+{\varphi }_{2}{d}_{n}\right)\cdot G\left({z}_{t}\right)+{\varepsilon }_{t}\end{array}$$

4 $$\Delta {\tilde{i }}_{t}=\theta {\beta }^{{^{\prime}}}{x}_{t-1}\cdot G\left({z}_{t}\right)+\left(\sum_{i=1}^{q}{\gamma }_{21,i}\Delta {s}_{t-i}+\sum_{i=1}^{q}{\gamma }_{22,i}\Delta {\tilde{i }}_{t-i}\right)\cdot G\left({z}_{t}\right)+\left({\varphi }_{1}{d}_{p}+{\varphi }_{2}{d}_{n}\right)\cdot G\left({z}_{t}\right)+{\varepsilon }_{t}$$

where all variables are defined as before. The lag length is chosen using the Likelihood-Ratio (LR) Test to select the most parsimonious specification which ensures that there is no serial correlation.

### Testing for Smooth Transition Nonlinearity

An important step prior to the estimation of the nonlinear model is performing nonlinearity tests. A suitable one in our case is a test for linearity against a STAR-type model. The null is that $\gamma =0$ versus $\gamma >0$, where $\gamma$ is the slope parameter, which indicates the smoothness of the transition from one regime to another. Teräsvirta and Yang (2014) report that Rao’s F statistic has better finite sample properties than other tests. The test statistic for the hypothesis $H_0:\theta=\theta_0$ is $RSS=S{\left({\theta }_{0}\right)}^{{^{\prime}}}{[I\left({\theta }_{0}\right)]}^{-1}S\left({\theta }_{0}\right)$, where $S$ is the score vector and $I$ is the Fisher information matrix. This test follows a chi-square distribution with r degrees of freedom, where r is the number of parameter vectors (Rao 1948). If linearity is rejected against a smooth transition nonlinearity type, one then also tests for the type of transition function. This is done by choosing a transition variable and then performing a test of the shape of the transition function. The test is based on a conventional STAR model, which can be expressed as follows:

5 $$\begin{array}{c}{y}_{t}={\pi }^{{^{\prime}}}{X}_{t}+F\left({z}_{t-d},\gamma ,c\right)+{\Theta }^{{^{\prime}}}{X}_{t}+{u}_{t}\end{array}$$

where ${X}_{t}=(1,{y}_{t-1},\dots ,{y}_{t-p})$ and $F\left({z}_{t-d},\gamma ,c\right)$ is the transition function, ${z}_{t-d}$ is the transition variable with lag $d$, $\gamma$ is the smoothness parameter and $c$ is the transition value. STAR-type models can either be logistic or exponential. The first order logistic transition function is:

6 $$\begin{array}{c}F\left({z}_{t-d},\gamma ,c\right)=\left[1-{exp}\left\{-\gamma {\left({z}_{t-d}-c\right)}^{2}\right\}\right]\end{array}$$

and the exponential transition function is:

7 $$\begin{array}{c}F\left({z}_{t-d},\gamma ,c\right)=\left[{\{1+{exp}(-\gamma \left({z}_{t-d}-c\right))\}}^{-1}-\frac{1}{2}\right]\end{array}$$

After the null of linearity is rejected one has to choose between a logistic and exponential transition function. For this purpose, Escribano and Jordá (2001) suggest a selection process based on the below auxiliary regression, which is a 4th order Taylor approximation of the generic transition function $F\left({z}_{t-d},\gamma ,c\right)$:

8 $$\begin{array}{c}{y}_{t}={\delta }_{0}+{\delta }_{1}^{{^{\prime}}}{\tilde{x }}_{t}+{\beta }_{1}^{{^{\prime}}}{\tilde{x }}_{t}{z}_{t-d}+{\beta }_{2}^{{^{\prime}}}{\tilde{x }}_{t}{z}_{t-d}^{2}+{\beta }_{3}^{{^{\prime}}}{\tilde{x }}_{t}{z}_{t-d}^{3}+{\beta }_{4}^{{^{\prime}}}{\tilde{x }}_{t}{z}_{t-d}^{4}+{\vartheta }_{3t}\end{array}$$

Using an F-test, the following hypotheses are tested:

$${H}_{{0}_{3}}:{\beta }_{3}^{{^{\prime}}}=0$$

$${H}_{{0}_{2}}:{\beta }_{2}^{{^{\prime}}}=0 | {\beta }_{3}^{{^{\prime}}}=0$$

$${H}_{{0}_{1}}:{\beta }_{1}^{{^{\prime}}}=0 | {\beta }_{2}^{{^{\prime}}}={\beta }_{3}^{{^{\prime}}}=0$$

Escribano and Jordá (2001) suggest the use of an F-test to test the null hypothesis ${H}_{{0}_{L}}:{\beta }_{2}^{{^{\prime}}}={\beta }_{4}^{{^{\prime}}}=0$ and to obtain the p-value for the test statistic ${F}_{L}$, and also to test the null hypothesis ${H}_{{0}_{L}}:{\beta }_{1}^{{^{\prime}}}={\beta }_{3}^{{^{\prime}}}=0$ and obtain the p-value for the test statistic ${F}_{E}$. If the p-value of ${F}_{E}$ is the minimum p-value, a logistic model should be selected; otherwise, the exponential model is more appropriate. We follow the method proposed by Escribano and Jordá (2001 – EJ henceforth) to select the most suitable transition function for each model.

### Misspecification Tests for Smooth Transition Models

Nonlinear smooth transition models can suffer from several types of misspecification. Eitrheim and Teräsvirta (1996) develop parametric testing procedures with desirable power properties to address these issues in smooth transition models. The first is a test of no additional nonlinearity, which is an LM-type test of the null of remaining nonlinearity against the alternative of no remaining nonlinearity. The second is a parameter constancy test, which is also an LM-type test. The third is an LM test of the serial independence of the error (Lukkonen and Teräsvirta 1988). In order to confirm the suitability of our choice of transition variable we also estimate STCVAR models which include alternative transitions variables, more precisely, the relative 30-day interest rate, the absolute change in the 30-day interest rate, the 30-day interest rate minus the overnight rate and the 10-year interest rate minus the overnight rate. To investigate in greater depth the role of central bank announcements, we also estimate a STCVAR model which excludes the announcement dummy variables.

## Data and Empirical Results

### Data Description

We use daily data from 1st January 2000 to 31st December 2020 for five inflation targeting countries (the UK, Canada, Australia, New Zealand and Sweden) and also for three non-inflation targeting economies (the US, the Euro-Area and Switzerland; Neumann and Von Hagen 2002). The nominal exchange rate series are obtained from the Pacific Exchange Rate Service database. The interest rate series and their sources are the following: for the UK the series used is the Bank of England Overnight London Interbank Offered Rate (LIBOR) based on the British Pound which is obtained from the Federal Reserve Bank of St Louis economic database; for Canada the series is the Bank of Canada Overnight Repo Rate taken from the Bank of Canada statistics database; for Australia it is the Reserve Bank of Australia Interbank Overnight Cash Rate retrieved from the Reserve Bank of Australia statistics database; for New Zealand it is the Reserve Bank of New Zealand Interbank Overnight Cash Rate reported in the Reserve Bank of New Zealand statistics database; for Sweden it is the Swedish Riksbank Deposit Rate from the Riksbank statistics database; for the US it is the Treasury Overnight London Interbank Offered Rate (LIBOR) based on the US Dollar, for Switzerland the Swiss National Bank Overnight London Interbank Offered Rate (LIBOR) based on the Swiss Franc, both series coming from the Federal Reserve Bank of St Louis economics database; finally, for the Euro-Area we use the European Central Bank EMU Convergence criteria daily interest rate series obtained from Eurostat. Central bank announcement dates are collected from the Bloomberg release calendars for individual central banks and include announcements of both positive and negative interest rate changes. The data for all 30-day interest rate series are obtained from Bloomberg. For the UK, the series is the 1-month LIBOR rate for the British pound; for Canada, the series is the 1-month Canadian banker acceptance rate; for Australia and New Zealand, the series are the 30-day interbank cash rate future contract; for Sweden, it is the 1-month interbank offered rate; for the US the 30-day Federal funds future rate; for the Euro-Area the 1-month EURIBOR rate; finally, for Switzerland the 1-month LIBOR for the Swiss franc. Daily changes are included in the model as a measure of changes in the expected interest rate over the following month. Data for the 10-year interest rates for all countries are also obtained from Bloomberg; specifically they are the daily rates on government bonds with 10-year maturity.

Exchange rates and interest rate differentials are defined in a consistent way, namely the domestic currency appears in the numerator and the foreign one in the denominator in the case of the former variable, whilst the latter variable is calculated as domestic minus foreign interest rates. For instance, CADAUD denotes the exchange rate constructed as domestic currency (in this case the Canadian dollar) units per unit of foreign currency (in this case the Australian dollar) and the interest differential is calculated as domestic interest rate (in this case the Canadian one) relative to the foreign interest rate (in this case the Australian one).

### Unit Root and Cointegration Tests

We first perform the DF-GLS and KPSS unit root tests on the nominal exchange rate and the interest rate differential series. The results of these tests are reported in Table 1 and confirm that all series are integrated of order $I(1)$.

**Table 1:** Unit Root Test Results

	**DF-GLS Test**			**KPSS Test**	
	**Level series**	**Differenced series**		**Level series**	**Differenced series**
**Nominal Exchange Rates**					
GBPCAD	-1.981	-15.484***		89.2***	0.042
GBPAUD	-1.906	-16.760***		103***	0.0676
GBPNZD	-2.616	-16.538***		68.8***	0.0315
GBPSEK	-2.314	-14.824***		94.7***	0.0326
CADAUD	-2.532	-13.089***		51.3***	0.0292
CADNZD	-2.741	-15.218***		32.2***	0.0282
CADSEK	-2.241	-15.186***		17.4***	0.0181
AUDNZD	-2.524	-14.633***		80.7***	0.0382
AUDSEK	-2.789	-13.267***		59.2***	0.0203
NZDSEK	-2.033	-15.562***		19***	0.0133
USDEUR	-1.553	-14.132***		149***	0.0675
USDCHF	-2.005	-15.993***		140***	0.029
EURCHF	-1.598	-16.754***		97.7***	0.0653
**Interest Rate Differentials**					
UK-Canada	-2.038	-17.027***		40.5***	0.0061
UK-Australia	-0.056	-16.351***		133***	0.0105
UK-New Zealand	-0.443	-17.102***		123***	0.0031
UK-Sweden	-1.007	-15.722***		61.7***	0.0587
Canada-Australia	-0.357	-16.056***		138***	0.0407
Canada-New Zealand	-0.463	-17.295***		108***	0.0084
Canada-Sweden	-0.921	-16.653***		64.5***	0.0219
Australia-New Zealand	-1.517	-17.606***		74.7***	0.0098
Australia-Sweden	-0.261	-16.425***		29.8***	0.0249
New Zealand-Sweden	-0.276	-17.566***		35.3***	0.119
US- Euro Area	-0.852	-12.248***		119***	0.0782
US-Switzerland	-1.811	-17.526***		116***	0.0019
Euro Area-Switzerland	-2.231	-16.243***		25.3***	0.0012
*** significant at 1% level					
DF-GLS: ${H}_{0}:$ variable contains a unit root ${H}_{1}:$ variable is stationary			KPSS: ${H}_{0}:$ variable is trend stationary ${H}_{1}:$ variable is not trend stationary		

Therefore, we proceed to test for cointegration between the series. The results of the Johansen cointegration trace and eigenvalue tests are reported in Table 2 and show that the cointegration rank is ${r}=1$, i.e. there exists a single cointegration relation in each case which can be interpreted as the UIP equilibrium.

**Table 2:** Johansen Trace and Eigenvalue Tests for Cointegration

	**Trace Test**		**Eigenvalue Test**	
	**Test 1**	**Test 2**	**Test 1**	**Test 2**
UK-Canada	0.0003***	0.4879	0.0001***	0.4879
UK-Australia	0.0129**	0.9323	0.0023***	0.9323
UK-New Zealand	0.0130**	0.4064	0.0103***	0.4064
UK-Sweden	0.0022**	0.2151	0.0012***	0.6755
Canada-Australia	0.0252**	0.5501	0.0149***	0.5501
Canada-New Zealand	0.0246**	0.5144	0.0014***	0.5186
Canada-Sweden	0.0364**	0.8298	0.0068***	0.2032
Australia-New Zealand	0.0006***	0.6791	0.0001***	0.6791
Australia-Sweden	0.0005***	0.5822	0.0019***	0.1831
New Zealand-Sweden	0.0185**	0.9249	0.0037***	0.9249
US-Euro Area	0.0057***	0.3192	0.0005***	0.9924
US-Switzerland	0.0000***	0.5672	0.0000***	0.5672
Euro Area-Switzerland	0.0000***	0.6506	0.0000***	0.6506
Trace Test: Test 1: ${H}_{0}: r=0; {H}_{1}: r=1$; 95% Critical value: 25.87 Test 2: ${H}_{0}: r\le 1; {H}_{1}: r=2$; 95% Critical value: 12.52		Eigenvalue Test: Test 1: ${H}_{0}: r=0; {H}_{1}: r=1$; 95% Critical value: 19.39 Test 2: ${H}_{0}: r\le 1; {H}_{1}: r=2$; 95% Critical value: 12.52		
$r$ denotes the cointegration rank and number of significant vectors. P-vales reported for all				

### Results for the Linear CVAR Model

Table 3 reports the results of LR tests to determine the optimal lag length for each CVAR model.

**Table 3:** Lag Selection in the CVAR Model

Lag	GBPCAD	GBPAUD	GBPNZD	GBPSEK	CADAUD	CADNZD	CADSEK
1	202.52	281.66	378.38	157.26	34.535	575.39	31.95
2	111.79	148.31	112.84	75.801	35.189	63.019	3.7235*
3	84.766*	90.054*	125.91	41.771	16.667	98.968*	1.0889
4	83.636	92.736	88.224	36.602	27.725	38.668	3.857
5	25.886	25.359	27.667	9.5262*	1.961*	16.924	0.749
6	44.641	59.112	58.974*	34.912	8.4577	9.7212	4.657
**Lag**	**AUDNZD**	**AUDSEK**	**NZDSEK**	**USDEUR**	**USDCHF**	**EURCHF**	
1	569.2	19.606	268.73	5.4953	789.68	791.48	
2	53.556	9.4629	11.58	28.972	243.08	212.93	
3	73.951*	4.4599*	33.226	3.1102*	100.29	79.738*	
4	64.427	16.419	16.652	14.305	263.16	278.17	
5	21.852	6.5165	9.84*	32.522	85.31*	333.98	
6	13.433	11.993	0.729	21.335	335.8	81.863	
Likelihood Ratio Test: sequential modified LR test statistic at 5% * indicates chosen lag at which there exists no serial correlation							

The estimation results for the linear Cointegrated Vector Autoregressive model are reported in Tables 4, 5 and 6. We find that a long-run relationship between the exchange rate and the interest rate differential exists in most cases. Since we have previously established that the exchange rate series are integrated of order I(1), they enter the cointegration relationship in the CVAR model in their levels, not their first differences as specified by the theoretical UIP relation. Therefore, while we find evidence for a long run equilibrium relationship between the exchange rate and the interest rate differential, it is not fully consistent with UIP.^1^The adjustment speed is low, with a maximum value of 1.7% for the AUDNZD exchange rate. These findings indicate that deviations from the equilibrium relationship between the exchange rate and the interest rate differential are highly persistent at a daily frequency. In the short run, there is no relation between the exchange rate and the interest rate differential. Central bank announcements of an interest rate decrease generally have a negative effect on the exchange rate (an appreciation) and the interest rate differential. There is no significant difference between inflation targeting countries and the other economies.

**Table 4:** Linear Cointegrated Vector Autoregressive Model Results for Non-Targeting Countries

	**USDEUR**		**USDCHF**		**EURCHF**	
**Independent Variables**	**Dependent Variables**					
	${\Delta {\varvec{s}}}_{{\varvec{t}}}$	${\Delta \stackrel{\sim }{{\varvec{i}}}}_{{\varvec{t}}}$	${\Delta {\varvec{s}}}_{{\varvec{t}}}$	${\Delta \stackrel{\sim }{{\varvec{i}}}}_{{\varvec{t}}}$	${\Delta {\varvec{s}}}_{{\varvec{t}}}$	${\Delta \stackrel{\sim }{{\varvec{i}}}}_{{\varvec{t}}}$
${\mu }_{0}$	0.00002	0.00021	0.00003	0.00039	0.00003	0.00017
	(0.00006)	(0.00025)	(0.00006)	(0.00181)	(0.00004)	(0.0019)
${\Delta s}_{t-1}$	0.00753	-0.047	0.0273**	0.00697	0.127***	0.801
	(0.0114)	(0.05)	(0.0114)	(0.325)	(0.0114)	(0.551)
${\Delta s}_{t-2}$	0.00628	0.0351	0.00788	-0.159	-0.0304***	0.118
	(0.0114)	(0.05)	(0.0114)	(0.325)	(0.0115)	(0.555)
${\Delta s}_{t-3}$	-0.00048	0.0286	-0.013	0.519	-0.0164	0.0502
	(0.0114)	(0.05)	(0.0114)	(0.324)	(0.0114)	(0.551)
${\Delta s}_{t-4}$			-0.0275**	0.129		
			(0.0114)	(0.324)		
${\Delta s}_{t-5}$			0.0067	0.383		
			(0.0114)	(0.324)		
${\Delta \tilde{i }}_{t-1}$	-0.00111	-0.0254**	0.00043	-0.444***	-0.00004	-0.365***
	(0.0026)	(0.0114)	(0.00039)	(0.0112)	(0.00024)	(0.0116)
${\Delta \tilde{i }}_{t-2}$	-0.00209	-0.0599***	-0.00004	-0.295***	0.00002	-0.201***
	(0.0026)	(0.0114)	(0.00042)	(0.0119)	(0.00025)	(0.012)
${\Delta \tilde{i }}_{t-3}$	0.00193	-0.0171	-0.00044	-0.234***	-0.00010	-0.100***
	(0.0026)	(0.0114)	(0.00042)	(0.0121)	(0.00024)	(0.0114)
${\Delta \tilde{i }}_{t-4}$			0.00004	-0.266***		
			(0.00042)	(0.0119)		
${\Delta \tilde{i }}_{t-5}$			-0.00017	-0.206***		
			(0.00039)	(0.0112)		
$\theta$	-0.00081*	0.00008	-0.00056*	0.0118	-0.00012	0.170***
	(0.00044)	(0.0019)	(0.00033)	(0.00925)	(0.00039)	(0.0186)
${d}_{p}$	0.00004	-0.0032**	0.00160***	-0.00561	0.00003	-0.00419
	(0.0003)	(0.0015)	(0.000491)	(0.014)	(0.00027)	(0.0131)
${d}_{n}$	0.00026	-0.0156***	0.00259***	-0.021	0.00259***	-0.00942
	(0.0009)	(0.0039)	(0.000815)	(0.0232)	(0.00049)	(0.0236)
* significant at 10% level, ** significant at 5% level, *** significant at 1% level. Standard errors in parentheses

**Table 5:** Linear Cointegrated Vector Autoregressive Model Results for Inflation Targeting Countries

	**GBPCAD**		**GBPAUD**		**CADNZD**		**AUDNZD**		**AUDSEK**	
**Independent Variables**	**Dependent Variables**									
	${\Delta {\varvec{s}}}_{{\varvec{t}}}$	${\Delta \stackrel{\sim }{{\varvec{i}}}}_{{\varvec{t}}}$	${\Delta {\varvec{s}}}_{{\varvec{t}}}$	${\Delta \stackrel{\sim }{{\varvec{i}}}}_{{\varvec{t}}}$	${\Delta {\varvec{s}}}_{{\varvec{t}}}$	${\Delta \stackrel{\sim }{{\varvec{i}}}}_{{\varvec{t}}}$	${\Delta {\varvec{s}}}_{{\varvec{t}}}$	${\Delta \stackrel{\sim }{{\varvec{i}}}}_{{\varvec{t}}}$	${\Delta {\varvec{s}}}_{{\varvec{t}}}$	${\Delta \stackrel{\sim }{{\varvec{i}}}}_{{\varvec{t}}}$
${\mu }_{0}$	0.00004	-0.0004	0.00002	0.000069	0.000058	0.000249	0.00004	-0.00018	-0.00004	-0.00007
	(0.00006)	(0.0004)	(0.00007)	(0.00039)	(0.00007)	(0.000286)	(0.000047)	(0.00026)	(0.00007)	(0.0002)
${\Delta s}_{t-1}$	0.00424	0.0819	-0.0106	-0.0918	-0.0106	-0.00956	-0.0152	0.0462	-0.0382***	-0.108***
	(0.0114)	(0.0801)	(0.0114)	(0.066)	(0.0114)	(0.0496)	(0.0114)	(0.0643)	(0.0114)	(0.0404)
${\Delta s}_{t-2}$	-0.0138	0.183**	0.00969	0.00939	-0.00838	-0.0449	-0.00554	-0.0786	0.0281**	-0.036
	(0.0114)	(0.0801)	(0.0114)	(0.066)	(0.0114)	(0.0496)	(0.0114)	(0.0642)	(0.0114)	(0.0404)
${\Delta s}_{t-3}$	0.015	0.113	-0.00827	0.0633	-0.00926	-0.015	-0.00888	-0.0633	-0.0246**	-0.0072
	(0.0114)	(0.0801)	(0.0114)	(0.066)	(0.0114)	(0.0495)	(0.0114)	(0.0642)	(0.0114)	(0.0404)
${\Delta \tilde{i }}_{t-1}$	0.00002	-0.193***	-0.00264	-0.231***	-0.00257	-0.297***	0.00152	-0.300***	0.00212	-0.00134
	(0.00162)	(0.0114)	(0.00197)	(0.0114)	(0.00258)	(0.0112)	(0.00201)	(0.0113)	(0.00323)	(0.0114)
${\Delta \tilde{i }}_{t-2}$	-0.00227	-0.134***	-0.00009	-0.161***	0.0019	-0.120***	-0.00211	-0.124***	0.00453	-0.00002
	(0.00163)	(0.0114)	(0.002)	(0.0115)	(0.00267)	(0.0116)	(0.00208)	(0.0117)	(0.00323)	(0.0114)
${\Delta \tilde{i }}_{t-3}$	0.00169	-0.101***	-0.0006	-0.107***	-0.00356	-0.110***	-0.00254	-0.148***	-0.000345	0.00005
	(0.00161)	(0.0113)	(0.00197)	(0.0114)	(0.00258)	(0.0112)	(0.002)	(0.0113)	(0.00323)	(0.0114)
$\theta$	0.000074	-0.0120***	-0.00144**	-0.00605*	-0.00129**	0.000702	-0.00198**	-0.0171***	-0.00237***	0.00246
	(0.00028)	(0.00196)	(0.00056)	(0.00325)	(0.000589)	(0.00255)	(0.000827)	(0.00465)	(0.000782)	(0.00277)
${d}_{p}$	0.000099	-0.00064	0.000237	-0.000258	-0.000462	0.00224*	-0.000511**	0.00104	0.000121	0.000118
	(0.00024)	(0.0017)	(0.000309)	(0.00179)	(0.00030)	(0.00131)	(0.000243)	(0.00136)	(0.000301)	(0.00106)
${d}_{n}$	0.000539	0.0366***	0.00201**	-0.0116**	-0.00147*	-0.0518***	-0.000161	0.0227***	0.00201***	-0.00244
	(0.00060)	(0.00421)	(0.000786)	(0.00454)	(0.000781)	(0.00339)	(0.000648)	(0.00365)	(0.000766)	(0.00271)
* significant at 10% level, ** significant at 5% level, *** significant at 1% level. Standard errors in parentheses

**Table 6:** Linear Cointegrated Vector Autoregressive Model Results for Inflation Targeting Countries

	**GBPNZD**		**GBPSEK**		**CADAUD**		**CADSEK**		**AUDSEK**	
**Independent Variables**	**Dependent Variables**									
	${\Delta {\varvec{s}}}_{{\varvec{t}}}$	${\Delta \stackrel{\sim }{{\varvec{i}}}}_{{\varvec{t}}}$	${\Delta {\varvec{s}}}_{{\varvec{t}}}$	${\Delta \stackrel{\sim }{{\varvec{i}}}}_{{\varvec{t}}}$	${\Delta {\varvec{s}}}_{{\varvec{t}}}$	${\Delta \stackrel{\sim }{{\varvec{i}}}}_{{\varvec{t}}}$	${\Delta {\varvec{s}}}_{{\varvec{t}}}$	${\Delta \stackrel{\sim }{{\varvec{i}}}}_{{\varvec{t}}}$	${\Delta {\varvec{s}}}_{{\varvec{t}}}$	${\Delta \stackrel{\sim }{{\varvec{i}}}}_{{\varvec{t}}}$
${\mu }_{0}$	0.00006	-0.00006	0.0000068	0.00001	0.00001	0.00043***	-0.00002	0.000354	-0.000074	-0.00003
	(0.00007)	(0.00045)	(0.00006)	(0.00044)	(0.00006)	(0.00015)	(0.00006)	(0.00026)	(0.00007)	(0.00034)
${\Delta s}_{t-1}$	0.00828	-0.0667	0.0149	-0.123	-0.0433***	-0.0317	0.0000722	-0.0874*	-0.0214*	-0.0799
	(0.0114)	(0.0739)	(0.0114)	(0.0845)	(0.0114)	(0.0285)	(0.0114)	(0.046)	(0.0114)	(0.055)
${\Delta s}_{t-2}$	-0.01	-0.0845	-0.0138	0.162*	0.0134	-0.0621**	-0.000795	0.00395	0.00314	-0.0636
	(0.0114)	(0.0738)	(0.0114)	(0.0845)	(0.0114)	(0.0285)	(0.0114)	(0.0459)	(0.0114)	(0.055)
${\Delta s}_{t-3}$	0.00468	0.0193	-0.0194*	-0.0087	-0.0355***	-0.0175			-0.0174	-0.0148
	(0.0114)	(0.0738)	(0.0114)	(0.0845)	(0.0114)	(0.0284)			(0.0114)	(0.0549)
${\Delta s}_{t-4}$	-0.0193*	-0.165**	-0.0199*	-0.0898	-0.0366***	-0.0182			-0.0346***	0.0102
	(0.0114)	(0.0738)	(0.0114)	(0.0845)	(0.0114)	(0.0284)			(0.0114)	(0.0549)
${\Delta s}_{t-5}$	-0.00404	-0.059	-0.0135	-0.148*	0.00254	-0.0118			0.00598	-0.0152
	(0.0114)	(0.0738)	(0.0114)	(0.0845)	(0.0114)	(0.0284)			(0.0114)	(0.0549)
${\Delta s}_{t-6}$	-0.00325	0.0122								
	(0.0114)	(0.0738)								
${\Delta \tilde{i }}_{t-1}$	-0.00286	-0.285***	0.00184	-0.167***	-0.00254	-0.0483***	-0.00755***	-0.0518***	0.00118	-0.191***
	(0.00177)	(0.0114)	(0.00154)	(0.0114)	(0.0043)	(0.0107)	(0.00278)	(0.0112)	(0.00237)	(0.0114)
${\Delta \tilde{i }}_{t-2}$	0.000159	-0.187***	0.000841	-0.115***	0.0214***	-0.0195*	0.00405	-0.0139	0.00557**	-0.0355***
	(0.00184)	(0.0119)	(0.00156)	(0.0115)	(0.0043)	(0.0107)	(0.00278)	(0.0112)	(0.00241)	(0.0116)
${\Delta \tilde{i }}_{t-3}$	0.000822	-0.178***	-0.00058	-0.0840***	0.00193	-0.0317***			-0.00128	-0.0691***
	(0.00185)	(0.012)	(0.00157)	(0.0116)	(0.00431)	(0.0108)			(0.00241)	(0.0116)
${\Delta \tilde{i }}_{t-4}$	-0.00167	-0.132***	-0.00105	-0.0689***	0.00778*	-0.0405***			-0.00299	-0.0288**
	(0.00185)	(0.0119)	(0.00156)	(0.0115)	(0.00431)	(0.0108)			(0.00241)	(0.0116)
${\Delta \tilde{i }}_{t-5}$	0.00369**	-0.0803***	-0.000323	-0.0257**	0.00473	-0.00773			0.0000498	-0.00703
	(0.00183)	(0.0118)	(0.00154)	(0.0114)	(0.00431)	(0.0107)			(0.00237)	(0.0114)
${\Delta \tilde{i }}_{t-6}$	-0.000434	-0.0873***								
	(0.00176)	(0.0114)								
$\theta$	-0.000731	-0.00442	-0.000956*	-0.00209	-0.00290***	0.00211	-0.00369***	0.00746*	-0.00179**	0.000657
	(0.000446)	(0.00288)	(0.000529)	(0.00391)	(0.000839)	(0.00209)	(0.000946)	(0.00381)	(0.000698)	(0.00336)
${d}_{p}$	0.0000431	0.00144	0.00033	-0.000196	0.000298	0.000014	0.00014	0.000156	0.000412	0.00094
	(0.000361)	(0.00233)	(0.000273)	(0.00202)	(0.000246)	(0.000614)	(0.000262)	(0.00106)	(0.000361)	(0.00174)
${d}_{n}$	0.00163*	-0.00763	0.000704	-0.0170***	-0.00335***	-0.0529***	0.000297	-0.0468***	0.00230***	-0.0141***
	(0.000867)	(0.0056)	(0.000638)	(0.00472)	(0.000664)	(0.00166)	(0.000649)	(0.00261)	(0.00085)	(0.00409)
* significant at 10% level, ** significant at 5% level, *** significant at 1% level. Standard errors in parentheses										

Table 7 reports tests of whether the individual variables in the model are at most I(1) or instead I(2) (Juselius 2017). The results show that the former is the case and therefore there can exist an I(0) cointegrating relationship linking them.

**Table 7:** Tests for Individual Variable Integration Order in the CVAR

	${{\varvec{s}}}_{{\varvec{t}}}$	${\stackrel{\sim }{{\varvec{i}}}}_{{\varvec{t}}}$
GBPCAD	0.1022	0.5813
GBPAUD	0.6773	0.5121
GBPNZD	0.9192	0.6204
GBPSEK	0.9788	0.8629
CADAUD	0.5311	0.9467
CADNZD	0.8139	0.4369
CADSEK	0.4815	0.2042
AUDNZD	0.4898	0.5529
AUDSEK	0.8103	0.9615
NZDSEK	0.8303	0.4896
USDEUR	0.8433	0.8369
USDCHF	0.3689	0.7363
EURCHF	0.1871	0.9140
Hypotheses: ${H}_{0}: variable\; is\; at\; most\; I\;(1)$ ${H}_{1}: variable\; is\; I\;(2)$ p-values reports for all		

Next we perform a series of diagnostic tests to establish whether the linear model is data congruent. These results are reported in Table 8 and show that none of the models suffer from serial correlation or parameter instability. However, given the finding of a low adjustment speed in the case of the linear models, we perform nonlinearity tests to see whether a nonlinear framework can provide stronger evidence for the existence of an equilibrium relationship between the exchange rate and the interest rate differential both in the short and the long run.

**Table 8:** Diagnostic Tests for the Linear Models

	**Lag**	**Breusch-Godfrey LM Test for serial correlation**	**Stability condition satisfied**
GBPCAD	3	0.51757	Stable
GBPAUD	3	0.37914	Stable
GBPNZD	6	0.22475	Stable
GBPSEK	5	0.10444	Stable
CADAUD	5	0.20724	Stable
CADNZD	3	0.05954	Stable
CADSEK	2	0.11597	Stable
AUDNZD	3	0.05554	Stable
AUDSEK	3	0.11153	Stable
NZDSEK	5	0.17796	Stable
USDEUR	3	0.28590	Stable
USDCHF	5	0.05062	Stable
EURCHF	3	0.11112	Stable
We use the Newey-West coefficient covariance matrix Breusch-Godfrey LM Test for serial correlation: ${H}_{0}:no\; serial\; correlation$ ${H}_{1}:serial\; correlation$ VAR test for eigenvalue stability conditions. ‘Stable’ means that all eigenvalues lie inside the unit circle and the model satisfies the stability conditions			

### Nonlinearity Tests and Results of the Smooth Transition Model

We test for smooth-transition type nonlinearity by means of the Rao F-test; the results are reported in Table 9. The null of linearity is rejected for all models, which confirms that the series exhibit nonlinearities of the smooth transition type. Next we use the EJ selection method for the most appropriate transition function. The results of this test are also included in Table 9 and show in each case whether an exponential or a logistic transition function respectively should be used. Table 10 displays instead the results of the LR test used to determine the lag length for each model.

**Table 9:** Linearity Test: Rao F-Test; Escribano-Jordá Test and Transition Function

		**Rao F-Test**	**Escribano-Jordá Test**	**Transition function**
GBPCAD	Exchange Rate Equation	0.0000***	0.0000***	Exponential
	Interest Rate Equation	0.0000***	0.0000***	Logistic
GBPAUD	Exchange Rate Equation	0.0000***	0.0000***	Exponential
	Interest Rate Equation	0.0000***	0.0000***	Logistic
GBPNZD	Exchange Rate Equation	0.0000***	0.0000***	Logistic
	Interest Rate Equation	0.0000***	0.0000***	Logistic
GBPSEK	Exchange Rate Equation	0.0000***	0.0000***	Exponential
	Interest Rate Equation	0.0000***	0.0000***	Logistic
CADAUD	Exchange Rate Equation	0.0000***	0.0000***	Exponential
	Interest Rate Equation	0.0000***	0.0000***	Logistic
CADNZD	Exchange Rate Equation	0.0000***	0.0000***	Exponential
	Interest Rate Equation	0.0000***	0.0000***	Logistic
CADSEK	Exchange Rate Equation	0.0000***	0.0000***	Logistic
	Interest Rate Equation	0.0000***	0.0000***	Exponential
AUDNZD	Exchange Rate Equation	0.0000***	0.0000***	Exponential
	Interest Rate Equation	0.0000***	0.0000***	Logistic
AUDSEK	Exchange Rate Equation	0.0000***	0.0000***	Exponential
	Interest Rate Equation	0.0000***	0.0000***	Exponential
NZDSEK	Exchange Rate Equation	0.0000***	0.0000***	Exponential
	Interest Rate Equation	0.0000***	0.0000***	Logistic
USDEUR	Exchange Rate Equation	0.0000***	0.0000***	Logistic
	Interest Rate Equation	0.0000***	0.0000***	Logistic
USDCHF	Exchange Rate Equation	0.0000***	0.0000***	Exponential
	Interest Rate Equation	0.0000***	0.0000***	Exponential
EURCHF	Exchange Rate Equation	0.0000***	0.0000***	Exponential
	Interest Rate Equation	0.0000***	0.0000***	Logistic
*** significant at 1%. P-values reported for both tests Rao-F Test: ${H}_{0}:linearity$ ${H}_{1}: smooth-transition\; nonlinearity$			Escribano-Jordá Test: *Logistic Transition Function:* ${H}_{{0}_{L}}:{\beta }_{2}^{^{\prime}}={\beta }_{4}^{^{\prime}}=0$ ${H}_{{1}_{L}}:{\beta }_{2}^{^{\prime}}\ne {\beta }_{4}^{^{\prime}}\ne 0$ *Exponential Transition Function:* ${H}_{{0}_{L}}:{\beta }_{1}^{^{\prime}}={\beta }_{3}^{^{\prime}}=0$ ${H}_{{1}_{L}}:{\beta }_{1}^{^{\prime}}\ne {\beta }_{3}^{^{\prime}}\ne 0$

**Table 10:** Lag Selection for the STCVAR Model

**Lag**	**GBPCAD**	**GBPAUD**	**GBPNZD**	**GBPSEK**	**CADAUD**	**CADNZD**	**CADSEK**
1	22.12	77.1	23.6	17.7	6.96	4.32	5.86*
2	19.48	6.14	16.16	6.52*	54.62	0.56	11.08
3	6.8*	32.4*	24.17	0.18	33.38*	9.32*	0.98
4	3.18	59.18	10.70*	3.88	4.74	2.7	3.66
5	4.06	25.12	8.724	2.46	0.56	9	3.08
6	22.12	77.1	23.6	17.7	6.96	4.32	5.86
**Lag**	**AUDNZD**	**AUDSEK**	**NZDSEK**	**USDEUR**	**USDCHF**	**EURCHF**	
1	14.04	129.84	16.28	5.5	6.62	7.46*	
2	3.96	13.86	13.12	19.38*	15.98*	4.98	
3	8.02*	31.14*	10.42*	42.7	8.7	97.74	
4	6.18	16.52	4.9	13.8	0.82	97.58	
5	8.5	8.56	0.06	18.4	93.62	6.82	
6	14.04	129.84	16.28	5.5	6.62	7.46	
Likelihood Ratio Test: sequential modified LR test statistic at 5% * indicates the chosen lag at which there exists no serial correlation							

The estimation results of the nonlinear model are reported in Tables 11 and 12 for the inflation targeting countries, and in Table 13 for the non-targeting ones. Unlike the linear model, the nonlinear one provides some evidence for the existence of a short-run relation between the exchange rate and the interest rate differentials in both regimes. The interest rate differential has a negative effect on the exchange rate in regime one, and a positive one in regime two, while in some countries the exchange rate affects negatively the interest rate differential in regime two. Both positive and negative central bank announcements now influence the exchange rate and the interest rate differential. Interestingly, the effect of central bank announcements on the exchange rate and the interest rate differential switches sign from one regime to the other, i.e. it appears to depend on market expectations of the interest rate. There is less evidence for a short-run relation between the exchange rate and the interest rate differential in non-targeting economies.

**Table 11:** Smooth Transition Cointegrated Vector Autoregressive Model Results for Inflation Targeting Countries

	**GBPCAD**		**GBPAUD**		**GBPNZD**		**GBPSEK**		**CADAUD**	
Independent Variables	Dependent Variables									
	${\Delta {\varvec{s}}}_{{\varvec{t}}}$	${\Delta \stackrel{\sim }{{\varvec{i}}}}_{{\varvec{t}}}$	${\Delta {\varvec{s}}}_{{\varvec{t}}}$	${\Delta \stackrel{\sim }{{\varvec{i}}}}_{{\varvec{t}}}$	${\Delta {\varvec{s}}}_{{\varvec{t}}}$	${\Delta \stackrel{\sim }{{\varvec{i}}}}_{{\varvec{t}}}$	${\Delta {\varvec{s}}}_{{\varvec{t}}}$	${\Delta \stackrel{\sim }{{\varvec{i}}}}_{{\varvec{t}}}$	${\Delta {\varvec{s}}}_{{\varvec{t}}}$	${\Delta \stackrel{\sim }{{\varvec{i}}}}_{{\varvec{t}}}$
**Regime 1**										
${\mu }_{0}$	0.0002	-0.052^***^	0.0003	-0.028^***^	-0.091^***^	-0.034^***^	-0.0002^*^	0.013	0.001^***^	-0.024^***^
	(0.0008)	(0.005)	(0.0006)	(0.0018)	(0.005)	(0.003)	(0.0001)	(0.028)	(0.0003)	(0.002)
${\Delta s}_{t-1}$	0.0127	0.557^*^	-0.007	-0.457^***^	-0.137	-0.245	-0.021	0.125^***^	0.256^***^	0.111
	(0.014)	(0.332)	(0.012)	(0.164)	(0.648)	(0.249)	(0.021)	(0.042)	(0.053)	(0.158)
${\Delta s}_{t-2}$	-0.0172	1.908^***^	-0.013	1.122^***^	0.143	0.404	0.036	-0.080	0.139^***^	0.740^***^
	(0.014)	(0.364)	(0.012)	(0.197)	(0.738)	(0.297)	(0.024)	(0.067)	(0.046)	(0.198)
${\Delta s}_{t-3}$	-0.0355	-0.890^**^	-0.005	-0.339^*^	0.742	-0.764^**^			0.243^***^	-5.170^***^
	(0.022)	(0.347)	(0.012)	(0.199)	(0.756)	(0.312)			(0.059)	(0.381)
${\Delta s}_{t-4}$					-0.359	-0.871^***^				
					(0.714)	(0.322)				
${\Delta \tilde{i }}_{t-1}$	-0.0018	-0.396^***^	-0.005^**^	-0.465^***^	-0.456^***^	-0.467^***^	0.002	0.124^***^	-0.067^***^	-0.842^***^
	(0.0020)	(0.041)	(0.002)	(0.027)	(0.080)	(0.038)	(0.003)	(0.019)	(0.020)	(0.247)
${\Delta \tilde{i }}_{t-2}$	-0.0003	-0.410^***^	0.001	-0.523^***^	-0.521^***^	-0.663^***^	-0.005	0.332^***^	0.049^***^	-0.537^***^
	(0.0019)	(0.044)	(0.002)	(0.032)	(0.099)	(0.057)	(0.003)	(0.020)	(0.013)	(0.060)
${\Delta \tilde{i }}_{t-3}$	0.0009	-0.104^**^	0.001	-0.141^***^	-0.345^***^	-0.229^***^			0.014	4.282^***^
	(0.002)	(0.044)	(0.002)	(0.033)	(0.101)	(0.047)			(0.017)	(0.665)
${\Delta \tilde{i }}_{t-4}$					-0.390^***^	-0.238^***^				
					(0.104)	(0.048)				
$\theta$	0.0006^**^	0.069^***^	-0.002^***^	0.203^***^	0.114^***^	0.169^***^	-0.0006	0.319^***^	0.009^**^	0.201^***^
	(0.0003)	(0.012)	(0.0006)	(0.015)	(0.039)	(0.021)	(0.0009)	(0.030)	(0.004)	(0.025)
${d}_{p}$	-0.0009	0.012	0.001^***^	-0.004	-0.030	-0.017^*^	0.0001	-0.119^***^	-0.002^**^	0.191^***^
	(0.004)	(0.008)	(0.0003)	(0.005)	(0.021)	(0.010)	(0.0004)	(0.012)	(0.001)	(0.023)
${d}_{n}$	-0.002^***^	0.011	-0.0001	-0.001	-0.048	-0.020	0.003^**^	-0.342	0.002	-0.053^***^
	(0.0008)	(0.011)	(0.0009)	(0.009)	(0.033)	(0.012)	(0.0013)	(0.881)	(0.002)	(0.015)
**Regime 2**										
${\mu }_{0}$	-0.002^**^	0.087^***^	-0.0002	0.050^***^	0.064^***^	0.058^***^	0.006^***^	-0.781^***^	-0.003^***^	0.024^***^
	(0.0010)	(0.008)	(0.0007)	(0.003)	(0.006)	(0.005)	(0.0002)	(0.028)	(0.0006)	(0.0019)
${\Delta s}_{t-1}$	-0.1914	-0.705	0.0873	0.786^***^	0.363	0.450	0.098^**^	-0.127^***^	-0.582^***^	-0.155
	(0.1425)	(0.552)	(0.070)	(0.277)	(0.704)	(0.409)	(0.038)	(0.042)	(0.070)	(0.0016)
${\Delta s}_{t-2}$	0.0645	-3.039^***^	1.087^***^	-1.908^***^	-0.088	-0.809	-0.140^***^	0.817^***^	-0.28^***^	-0.801^***^
	(0.132)	(0.604)	(0.311)	(0.330)	(0.786)	(0.496)	(0.043)	(0.068)	(0.093)	(0.002)
${\Delta s}_{t-3}$	0.848^***^	1.734^***^	-0.231^**^	0.779^**^	-0.681	1.421^***^			-0.545^***^	5.177^***^
	(0.186)	(0.577)	(0.115)	(0.337)	(0.803)	(0.514)			(0.092)	(0.038)
${\Delta s}_{t-4}$					0.498	1.309^**^				
					(0.765)	(0.542)				
${\Delta \tilde{i }}_{t-1}$	0.0188^*^	0.358^***^	0.109^***^	0.476^***^	0.254^***^	0.349^***^	-0.0007	-0.120^***^	0.125^***^	0.806^***^
	(0.011)	(0.065)	(0.041)	(0.048)	(0.092)	(0.064)	(0.004)	(0.019)	(0.037)	(0.002)
${\Delta \tilde{i }}_{t-2}$	-0.029^***^	0.504^***^	0.019	0.739^***^	0.432^***^	0.904^***^	0.012^**^	-0.328^***^	-0.069^**^	0.522^***^
	(0.011)	(0.070)	(0.044)	(0.056)	(0.108)	(0.093)	(0.005)	(0.020)	(0.030)	(0.061)
${\Delta \tilde{i }}_{t-3}$	0.016	0.013	-0.095^**^	0.092	0.234^**^	0.153^*^			-0.023	-4.31^***^
	(0.018)	(0.072)	(0.044)	(0.057)	(0.110)	(0.079)			(0.033)	(0.067)
${\Delta \tilde{i }}_{t-4}$					0.343^***^	0.257^***^				
					(0.112)	(0.080)				
$\theta$	0.0006	-0.133^***^	0.005	-0.379^***^	-0.174^***^	-0.298^***^	-0.0013	-0.323^***^	-0.02^***^	-0.200^***^
	(0.003)	(0.019)	(0.006)	(0.024)	(0.041)	(0.033)	(0.002)	(0.030)	(0.0073)	(0.026)
${d}_{p}$	0.0033	-0.018	-0.042^***^	0.012	-0.017	0.037^**^	0.0008	0.0011	0.003	-0.191^***^
	(0.005)	(0.014)	(0.014)	(0.009)	(0.023)	(0.016)	(0.0010)	(0.012)	(0.002)	(0.023)
${d}_{n}$	-0.017^***^	-0.017	-0.013	0.007	0.056	0.027	-0.007^***^	0.098	-0.005	0.059^***^
	(0.0056)	(0.019)	(0.010)	(0.015)	(0.041)	(0.020)	(0.0019)	(0.885)	(0.004)	(0.016)

* significant at 10% level, ** significant at 5% level, *** significant at 1% level. Standard errors in parentheses

**Table 12:** Smooth Transition Cointegrated Vector Autoregressive Model Results for Inflation Targeting Countries

	**CADNZD**		**CADSEK**		**AUDNZD**		**AUDSEK**		**NZDSEK**	
Independent Variables	Dependent Variables									
	${\Delta {\varvec{s}}}_{{\varvec{t}}}$	${\Delta \stackrel{\sim }{{\varvec{i}}}}_{{\varvec{t}}}$	${\Delta {\varvec{s}}}_{{\varvec{t}}}$	${\Delta \stackrel{\sim }{{\varvec{i}}}}_{{\varvec{t}}}$	${\Delta {\varvec{s}}}_{{\varvec{t}}}$	${\Delta \stackrel{\sim }{{\varvec{i}}}}_{{\varvec{t}}}$	${\Delta {\varvec{s}}}_{{\varvec{t}}}$	${\Delta \stackrel{\sim }{{\varvec{i}}}}_{{\varvec{t}}}$	${\Delta {\varvec{s}}}_{{\varvec{t}}}$	${\Delta \stackrel{\sim }{{\varvec{i}}}}_{{\varvec{t}}}$
**Regime 1**										
${\mu }_{0}$	0.005^***^	-0.0002	0.00008	0.0007^**^	-0.156^***^	-0.034^***^	-0.0002	0.025	-0.0006	0.010
	(0.0002)	(0.0003)	(0.0001)	(0.0003)	(0.006)	(0.002)	(0.0009)	(0.446)	(0.0007)	(0.009)
${\Delta s}_{t-1}$	0.034^*^	-0.0025	0.0017	0.0138	0.027^***^	-0.060	-0.035^**^	-0.066	-0.0085	0.246
	(0.021)	(0.050)	(0.0135)	(0.0555)	(0.001)	(0.437)	(0.015)	(98.843)	(0.013)	(1.413)
${\Delta s}_{t-2}$	-0.025	-0.0337			0.092	-0.098	-0.129^***^	-0.120	-0.014	0.446
	(0.020)	(0.050)			(1.159)	(0.421)	(0.039)	(112.16)	(0.012)	(1.418)
${\Delta s}_{t-3}$	0.026	-0.013			-0.016	-0.088	-0.012	1.46	-0.010	0.255
	(0.021)	(0.050)			(1.357)	(0.419)	(0.017)	(132.23)	(0.012)	(1.410)
${\Delta \tilde{i }}_{t-1}$	-0.0008	-0.279^***^	-0.009^***^	-0.051^***^	0.096	-0.206^***^	0.0004	0.076	-0.0013	0.470
	(0.004)	(0.011)	(0.0034)	(0.015)	(0.271)	(0.076)	(0.005)	(108.88)	(0.003)	(0.296)
${\Delta \tilde{i }}_{t-2}$	0.0014	-0.115^***^			0.071	-0.016	0.007	-0.146^***^	0.0047	0.613^**^
	(0.004)	(0.012)			(0.411)	(0.076)	(0.004)	(0.018)	(0.003)	(0.309)
${\Delta \tilde{i }}_{t-3}$	0.003	-0.100^***^			0.713^**^	-0.067	-0.081^**^	-1.141^***^	0.0004	0.444
	(0.004)	(0.011)			(0.332)	(0.072)	(0.038)	(0.015)	(0.002)	(0.293)
$\theta$	-0.002^**^	0.0021	-0.001	-0.016^**^	0.067	0.100^***^	-0.001	0.136	-0.0015	0.006
	(0.001)	(0.003)	(0.0013)	(0.0067)	(0.115)	(0.011)	(0.001)	(8.801)	(0.0008)	(0.086)
${d}_{p}$	0.0009	0.0014	-0.0002	0.0011	-0.041	0.023^**^	0.0005	0.010	0.0002	-0.02
	(0.0005)	(0.001)	(0.0003)	(0.0013)	(0.032)	(0.009)	(0.0005)	(6.619)	(0.0004)	(0.045)
${d}_{n}$	-0.004^***^	-0.012^***^	0.003^***^	0.025^***^	0.121^**^	-0.035	0.0009	-0.095	0.0029^**^	0.052
	(0.0013)	(0.004)	(0.0008)	(0.0032)	(0.055)	(0.028)	(0.0010)	(5.576)	(0.0011)	(0.108)
**Regime 2**										
${\mu }_{0}$	-0.002^***^	0.016^***^	-0.0004^**^	-0.014^***^	0.015^**^	0.038^***^	-0.0004	-0.016	0.0004^**^	0.010
	(0.0005)	(0.003)	(0.0002)	(0.0031)	(0.0066)	(0.005)	(0.0006)	(0.437)	(0.0002)	(0.014)
${\Delta s}_{t-1}$	-0.167^***^	-0.046	-0.0083	-1.192^***^	-0.030	-1.437	-0.020	0.067	-0.055^**^	-0.120^***^
	(0.053)	(0.297)	(0.028)	(0.355)	(1.197)	(1.022)	(0.073)	(99.68)	(0.027)	(0.0163)
${\Delta s}_{t-2}$	0.059	-0.050			-1.039	-0.387	1.426^***^	0.121	0.115^***^	-0.391^***^
	(0.066)	(0.338)			(1.177)	(1.197)	(0.195)	(112.97)	(0.032)	(0.0533)
${\Delta s}_{t-3}$	-0.141^**^	-0.834^**^			0.154	-0.295	-0.130	-1.07^***^	-0.049	-0.138^**^
	(0.066)	(0.330)			(1.376)	(1.236)	(0.107)	(133.20)	(0.033)	(0.0189)
${\Delta \tilde{i }}_{t-1}$	-0.009	-0.97^***^	0.011^*^	0.060	-0.256	-0.454^**^	0.0211	-0.076	0.010^*^	0.176^***^
	(0.014)	(0.068)	(0.006)	(0.052)	(0.275)	(0.197)	(0.037)	(109.35)	(0.006)	(0.0240)
${\Delta \tilde{i }}_{t-2}$	-0.001	-0.152^**^			-0.846^**^	-0.653^***^	-0.013	0.158	0.002	-0.279^**^
	(0.013)	(0.060)			(0.414)	(0.245)	(0.012)	(0.180)	(0.005)	(0.0379)
${\Delta \tilde{i }}_{t-3}$	-0.024	-0.058			-0.819^**^	-0.744^***^	0.886^**^	1.158	-0.016^*^	-0.420^***^
	(0.013)	(0.056)			(0.333)	(0.266)	(0.448)	(1501.1)	(0.009)	(0.0572)
$\theta$	0.004	0.100^***^	-0.011^***^	-0.425^***^	0.034	-0.330^***^	-0.016^**^	-0.378^***^	-0.002	-0.383^***^
	(0.004)	(0.024)	(0.003)	(0.069)	(0.116)	(0.029)	(0.008)	(0.088)	(0.002)	(0.0522)
${d}_{p}$	-0.0016	0.060^***^	-0.0004	0.011	0.028	0.015	-0.002	-0.010	-0.001	-0.043
	(0.002)	(0.008)	(0.0007)	(0.014)	(0.033)	(0.023)	(0.004)	(6.714)	(0.0009)	(0.0592)
${d}_{n}$	0.009^***^	-0.12^***^	-0.0012	-0.049^***^	-0.131^**^	-0.025	0.004	0.097	-0.003	0.011^***^
	(0.003)	(0.014)	(0.0014)	(0.011)	(0.057)	(0.048)	(0.004)	(5.502)	(0.002)	(0.002)

* significant at 10% level, ** significant at 5% level, *** significant at 1% level. Standard errors in parentheses

**Table 13:** Smooth Transition Cointegrated Vector Autoregressive Model Results for Non-Targeting Economies

	**USDEUR**		**USDCHF**		**EURCHF**	
Independent Variables	Dependent Variables					
	${\Delta {\varvec{s}}}_{{\varvec{t}}}$	${\Delta \stackrel{\sim }{{\varvec{i}}}}_{{\varvec{t}}}$	${\Delta {\varvec{s}}}_{{\varvec{t}}}$	${\Delta \stackrel{\sim }{{\varvec{i}}}}_{{\varvec{t}}}$	${\Delta {\varvec{s}}}_{{\varvec{t}}}$	${\Delta \stackrel{\sim }{{\varvec{i}}}}_{{\varvec{t}}}$
**Regime 1**						
${\mu }_{0}$	0.0001	0.0001	0.0002	-0.0012	-0.00005	0.0016
	(0.0002)	(0.0002)	(0.0002)	(0.0030)	(0.00005)	(0.0019)
${\Delta s}_{t-1}$	0.073^***^	0.0022	-0.087^*^	-0.0829	-0.0038	0.6241
	(0.0278)	(0.0454)	(0.0346)	(0.6158)	(0.0220)	(0.6668)
${\Delta s}_{t-2}$	-0.026	0.077^*^	-0.029	-0.236		
	(0.0316)	(0.0454)	(0.0271)	(0.4513)		
${\Delta \tilde{i }}_{t-1}$	-0.009	0.049^***^	0.0006	0.0112	-0.0003	-0.2498^***^
	(0.0059)	(0.0110)	(0.0007)	(0.0397)	(0.00032)	(0.0113)
${\Delta \tilde{i }}_{t-2}$	-0.008	-0.103^***^	0.0001	0.0119		
	(0.0055)	(0.0106)	(0.0007)	(0.0296)		
$\theta$	-0.0003	-0.0004	-0.0009	-0.032^***^	0.00014	-0.0764^***^
	(0.0002)	(0.0004)	(0.0007)	(0.0042)	(0.00015)	(0.0053)
${d}_{p}$	0.0025^***^	-0.0010	0.0034^***^	-0.025	-0.00057	-0.0095
	(0.0009)	(0.0013)	(0.0013)	(0.0363)	(0.00036)	(0.0137)
${d}_{n}$	0.0041^***^	-0.024^***^	0.0002	0.0283	0.00025	-0.0177
	(0.0016)	(0.0036)	(0.0016)	(0.070)	(0.0008)	(0.0245)
**Regime 2**						
${\mu }_{0}$	-0.0001	-0.476	0.0009^**^	0.0007	0.00030^**^	-0.0627^***^
	(0.0002)	(0.398)	(0.0004)	(0.0038)	(0.00013)	(0.0102)
${\Delta s}_{t-1}$	-0.110^***^	-0.880	0.244^***^	0.267	0.2617^***^	-0.768
	(0.0398)	(68.548)	(0.0461)	(0.7336)	(0.0326)	(1.3224)
${\Delta s}_{t-2}$	0.047	-0.628	0.089	0.296		
	(0.0456)	(66.942)	(0.0584)	(0.6625)		
${\Delta \tilde{i }}_{t-1}$	0.012	-1.775	-0.0004	-0.504^***^	0.00089	-0.1999^***^
	(0.0076)	(1.5558)	(0.0014)	(0.0419)	(0.0006)	(0.0429)
${\Delta \tilde{i }}_{t-2}$	0.011	-1.40	-0.0007	-0.276^***^		
	(0.0076)	(1.1302)	(0.0014)	(0.0322)		
$\theta$	0.0004	-0.209^***^	0.0009	-0.132^***^	-0.0008^**^	-0.1815^***^
	(0.0003)	(0.016)	(0.0016)	(0.0050)	(0.00035)	(0.0264)
${d}_{p}$	-0.006^***^	-0.660	-0.008^***^	0.0203	0.0019^**^	0.1779^***^
	(0.0013)	(0.6038)	(0.0023)	(0.0396)	(0.0008)	(0.0496)
${d}_{n}$	-0.005^**^	1.470	0.0041^*^	-0.0284	-0.00001	0.1198
	(0.0022)	(0.9988)	(0.0025)	(0.070)	(0.0012)	(0.1135)
* significant at 10% level, ** significant at 5% level, *** significant at 1% level. Standard errors in parentheses						

Table 14 provides information about the transition function, specifically the transition parameter $c$ and the smoothness parameter $\gamma$, and also reports the estimates of the $\theta$ coefficient (the speed of adjustment) for the two regimes—this the optimal number of regimes which is selected in all cases by using as a criterion the minimum sum of squared residuals. Graphs for the corresponding transition functions are included in the Appendix Figs. 1, 2, 3, 4, 5, 6, 7, 8, 9, 10, 11, 12, 13, 14, 15, 16, 17, 18, 19, 20, 21, 22, 23, 24, 25, 26.

**Table 14:** Smooth Transition Model Regimes

		**Regimes**		**Transition**	
	Equation	Regime 1: $\theta$	Regime 2: $\theta \bullet {G}_{t}$	$c$	$\gamma$
GBPCAD	${\Delta {\varvec{s}}}_{{\varvec{t}}}$	0.0006^**^	0.0006	-0.055868	14.78672
	${\Delta \stackrel{\sim }{{\varvec{i}}}}_{{\varvec{t}}}$	0.069^***^	-0.133^***^	-0.016814	22.65341
GBPAUD	${\Delta {\varvec{s}}}_{{\varvec{t}}}$	-0.002^***^	0.005	-0.005186	13.16741
	${\Delta \stackrel{\sim }{{\varvec{i}}}}_{{\varvec{t}}}$	0.203^***^	-0.379^***^	-0.006006	45.75886
GBPNZD	${\Delta {\varvec{s}}}_{{\varvec{t}}}$	0.114^***^	-0.174^***^	-0.012500	62.36919
	${\Delta \stackrel{\sim }{{\varvec{i}}}}_{{\varvec{t}}}$	0.169^***^	-0.298^***^	-0.012345	31.67953
GBPSEK	${\Delta {\varvec{s}}}_{{\varvec{t}}}$	-0.0006	-0.0013	0.020142	65.02806
	${\Delta \stackrel{\sim }{{\varvec{i}}}}_{{\varvec{t}}}$	0.319^***^	-0.323^***^	-0.272267	66.07738
CADAUD	${\Delta {\varvec{s}}}_{{\varvec{t}}}$	0.009^**^	-0.02^***^	-0.173465	21.68449
	${\Delta \stackrel{\sim }{{\varvec{i}}}}_{{\varvec{t}}}$	0.201^***^	-0.200^***^	-0.192480	15.45625
CADNZD	${\Delta {\varvec{s}}}_{{\varvec{t}}}$	-0.002^**^	0.004	-0.050491	85.06376
	${\Delta \stackrel{\sim }{{\varvec{i}}}}_{{\varvec{t}}}$	0.0021	0.100^***^	0.081161	18.91247
CADSEK	${\Delta {\varvec{s}}}_{{\varvec{t}}}$	-0.001	-0.011^***^	0.009453	42.40906
	${\Delta \stackrel{\sim }{{\varvec{i}}}}_{{\varvec{t}}}$	-0.016^**^	-0.425^***^	0.047907	16.40401
AUDNZD	${\Delta {\varvec{s}}}_{{\varvec{t}}}$	0.067	0.034	-0.088433	88.81159
	${\Delta \stackrel{\sim }{{\varvec{i}}}}_{{\varvec{t}}}$	0.100^***^	-0.330^***^	0.014948	46.41960
AUDSEK	${\Delta {\varvec{s}}}_{{\varvec{t}}}$	-0.001	-0.016^**^	-0.107431	8.321685
	${\Delta \stackrel{\sim }{{\varvec{i}}}}_{{\varvec{t}}}$	0.136	-0.378^***^	0.135389	2.311828
NZDSEK	${\Delta {\varvec{s}}}_{{\varvec{t}}}$	-0.0015	-0.002	0.010824	13.86116
	${\Delta \stackrel{\sim }{{\varvec{i}}}}_{{\varvec{t}}}$	0.006	-0.383^***^	2.131533	31.05755
USDEUR	${\Delta {\varvec{s}}}_{{\varvec{t}}}$	-0.0003	0.0004	-0.001864	34.44399
	${\Delta \stackrel{\sim }{{\varvec{i}}}}_{{\varvec{t}}}$	-0.0004	-0.209^***^	0.162205	26.28246
USDCHF	${\Delta {\varvec{s}}}_{{\varvec{t}}}$	-0.0009	0.0009	-0.018740	12.51957
	${\Delta \stackrel{\sim }{{\varvec{i}}}}_{{\varvec{t}}}$	-0.032^***^	-0.132^***^	0.000023	1.940000
EURCHF	${\Delta {\varvec{s}}}_{{\varvec{t}}}$	0.00014	-0.0008^**^	0.003511	67.78872
	${\Delta \stackrel{\sim }{{\varvec{i}}}}_{{\varvec{t}}}$	-0.0764^***^	-0.1815^***^	0.016260	17.30912
Transition variable ${z}_{t}$: change in the 30-day interest rate $\theta$ is the speed of adjustment parameter in regime 1; $\theta \bullet {G}_{t}$ is the speed of adjustment parameter in regime 2; c is the transition value, which indicates where the transition takes place; and $\gamma$ is the smoothness parameter which indicates the speed of transition					

It can be seen that the adjustment speed in the nonlinear model is substantially faster than in the linear one: between 10 and 43% of any deviations from the equilibrium is corrected within one day, which means that a nonlinear framework provides stronger evidence than a linear one in support of a long-run equilibrium relationship between the exchange rate and the interest rate differential. While the adjustment occurs in both the interest rate and the exchange rate equations, the speed is substantially faster in the case of the former. These findings imply that it is the interest rate differential (rather than the exchange rate) which adjusts to restore the equilibrium. The adjustment is particularly fast in regime two, i.e. when the change in the expected interest rate exceeds the transition value $c$. This suggests that the equilibrium relationship between the exchange rate and the interest rate differential generally holds better when interest rates are expected to increase. Evidence for error-increasing behaviour could be found in only one exchange rate equation (GBPNZD) and in regime 1 for some interest rate equations (for instance GBPAUD, GBPNZD, GBPSEK, CADAUD, AUDNZD). The positive coefficient in regime one in some equations indicates that deviations from the long-run equilibrium relationship between the exchange rate and the interest rate differential are persistent when the market expects the interest rate to fall in the near future. On average the non-targeting economies seem to be characterised by a lower adjustment speed than the inflation targeting ones, which suggests that interest rate expectations play a more important role in the adjustment towards the long-run equilibrium under inflation targeting. On the whole, the results in Table 15 indicate that the system moves back towards its long-run equilibrium through adjustments in the interest rate equation, but only when the market expects the central bank to adopt a contractionary monetary policy stance by raising the interest rate in the near future.

**Table 15:** Misspecification Tests for the Nonlinear STCVAR Models

	**Lag**	**Serial Independence**	**No remaining nonlinearity**	**Parameter constancy**
GBPCAD	3	0.5916	0.1141	0.0531
GBPAUD	3	0.9698	0.1629	0.1800
GBPNZD	4	0.1462	0.7581	0.1623
GBPSEK	2	0.6140	0.1560	0.3158
CADAUD	3	0.5677	0.1083	0.4600
CADNZD	3	0.9876	0.7039	0.1369
CADSEK	1	0.7790	0.8870	0.9510
AUDNZD	3	0.7638	0.6959	0.0762
AUDSEK	3	0.7067	0.4491	0.3835
NZDSEK	3	0.2819	0.0704	0.1590
USDEUR	2	0.3147	0.2530	0.1393
USDCHF	2	0.9187	0.2895	0.1517
EURCHF	1	0.5752	0.2070	0.2442
Lagrange Multiplier (LM) Test of serial correlation: ${H}_{0}:no\; serial\; correlation$ ${H}_{1}:serial\; correlation$ Lagrange Multiplier (LM) test of no remaining nonlinearity:${H}_{0}:no\; remaining\; nonlinearity$ ${H}_{1}:remaining\; nonlinearity$			Lagrange Multiplier (LM) test of parameter constancy: ${H}_{0}:parameter\; constancy$ ${H}_{1}:no\; parameter\; constancy$ P-values reported for all tests	

Finally, to check the adequacy of the nonlinear STCVAR model we conduct Lagrange Multiplier (LM) Tests of serial correlation, of no remaining nonlinearity and of parameter constancy. The test statistics are reported in Table 15 and confirm the data congruency of the nonlinear specification. In particular, there is no evidence of an impact of the recent COVID-19 pandemic, which is known to have affected other financial markets (e.g., Salisu and Vo 2020).

To assess the importance of the announcement dummies we compare the nonlinear STCVAR model with one from which the central bank announcement dummies are excluded. These results are reported in Tables 16, 17 and 18 and indicate that the adjustment speed tends to be lower when excluding the dummies from the model. Therefore accounting for central bank announcements of changes in the interest rate therefore seems to be important for understanding how interest rate expectations influence the adjustment towards the equilibrium relationship between the exchange rate and the interest rate differential.

**Table 16:** Smooth Transition Cointegrated Vector Autoregressive Model Results excluding Dummy Variables for Inflation Targeting Countries

	**GBPCAD**		**GBPAUD**		**GBPNZD**		**GBPSEK**		**CADAUD**	
Independent Variables	Dependent Variables									
	${\Delta {\varvec{s}}}_{{\varvec{t}}}$	${\Delta \stackrel{\sim }{{\varvec{i}}}}_{{\varvec{t}}}$	${\Delta {\varvec{s}}}_{{\varvec{t}}}$	${\Delta \stackrel{\sim }{{\varvec{i}}}}_{{\varvec{t}}}$	${\Delta {\varvec{s}}}_{{\varvec{t}}}$	${\Delta \stackrel{\sim }{{\varvec{i}}}}_{{\varvec{t}}}$	${\Delta {\varvec{s}}}_{{\varvec{t}}}$	${\Delta \stackrel{\sim }{{\varvec{i}}}}_{{\varvec{t}}}$	${\Delta {\varvec{s}}}_{{\varvec{t}}}$	${\Delta \stackrel{\sim }{{\varvec{i}}}}_{{\varvec{t}}}$
**Regime 1**										
${\mu }_{0}$	0.0002^**^	-0.050^***^	0.0014^***^	-0.028^***^	-0.913^***^	-0.035^***^	-0.0002	0.4089^***^	0.0014^***^	-0.026^***^
	(0.00008)	(0.0050)	(0.0003)	(0.0018)	(0.0052)	(0.0035)	(0.0001)	(0.0203)	(0.0003)	(0.0018)
${\Delta s}_{t-1}$	0.0016	0.552^*^	0.0188	-0.450^***^	-0.150	-0.237	-0.040	1.7184	0.2627^***^	0.0542
	(0.014)	(0.329)	(0.023)	(0.164)	(0.648)	(0.250)	(0.027)	(2.923)	(0.054)	(0.151)
${\Delta s}_{t-2}$	-0.0219	1.874^***^	-0.135^***^	1.122^***^	0.091	0.401	0.059	-2.619	0.137^***^	0.425^**^
	(0.014)	(0.359)	(0.034)	(0.197)	(0.737)	(0.299)	(0.032)	(2.995)	(0.0469)	(0.191)
${\Delta s}_{t-3}$	-0.033	-0.887^***^	0.102^***^	-0.333^*^	0.686	-0.769^**^			0.2518^***^	-4.943^***^
	(0.021)	(0.344)	(0.032)	(0.198)	(0.754)	(0.315)			(0.061)	(0.364)
${\Delta s}_{t-4}$					-0.372	-0.927^***^				
					(0.714)	(0.325)				
${\Delta \tilde{i }}_{t-1}$	-0.0009	-0.390^***^	-0.007^*^	-0.465^***^	-0.459^***^	-0.471^***^	0.002	1.686^***^	-0.076^***^	-0.751^***^
	(0.0019)	(0.040)	(0.004)	(0.027)	(0.080)	(0.038)	(0.0032)	(0.374)	(0.020)	(0.187)
${\Delta \tilde{i }}_{t-2}$	0.0004	-0.398^***^	0.003	-0.523^***^	-0.545^***^	-0.674^***^	-0.006*	-0.621^*^	0.0518^***^	-0.504^***^
	(0.002)	(0.043)	(0.0044)	(0.032)	(0.097)	(0.057)	(0.0037)	(0.338)	(0.0137)	(0.058)
${\Delta \tilde{i }}_{t-3}$	0.002	-0.107^**^	-0.002	-0.141^***^	-0.364^***^	-0.234^***^			0.0150	4.596^***^
	(0.002)	(0.043)	(0.005)	(0.033)	(0.101)	(0.047)			(0.017)	(0.614)
${\Delta \tilde{i }}_{t-4}$					-0.413^***^	-0.257^***^				
					(0.103)	(0.048)				
$\theta$	0.00002	0.067^***^	-0.004^***^	0.202^***^	0. 114^***^	0.170^***^	-0.0006	-0.375^**^	0.010^**^	0.141
	(0.0003)	(0.012)	(0.0015)	(0.015)	(0.039)	(0.021)	(0.001)	(0.162)	(0.0041)	(0.024)
**Regime 2**										
${\mu }_{0}$	-0.0019	0.085^***^	-0.004***	0.051^***^	0.064^***^	0.059^***^	0.0005^**^	0.1035^***^	-0.0029^***^	0.027^***^
	(0.0007)	(0.0073)	(0.0006)	(0.0029)	(0.0054)	(0.0053)	(0.0002)	(0.024)	(0.0006)	(0.0018)
${\Delta s}_{t-1}$	0.0107	-0.6971	-0.0763	0.774^***^	0.375	0.429	0.110^***^	-2.098	-0.5821^***^	-0.103
	(0.1039)	(0.5489)	(0.0611)	(0.2759)	(0.705)	(0.405)	(0.0047)	(3.606)	(0.071)	(0.1535)
${\Delta s}_{t-2}$	0.1204	-3.004^***^	0.4687^***^	-1.9037^***^	-0.0369	-0.796	-0.1497^***^	4.203	-0.265^**^	-0.479^**^
	0.1056	(0.599)	(0.083)	(0.330)	(0.786)	(0.492)	(0.0052)	(3.628)	(0.0936)	(0.194)
${\Delta s}_{t-3}$	0.666^***^	1.734^***^	-0.365^***^	0.767^**^	-0.608	1.408^***^			-0.5498^***^	4.948^***^
	(0.141)	(0.575)	(0.085)	(0.337)	(0.801)	(0.512)			(0.0935)	(0.365)
${\Delta s}_{t-4}$					0.559	1.374^**^				
					(0.764)	(0.537)				
${\Delta \tilde{i }}_{t-1}$	0.0070	0.351^***^	0.015	0.474^***^	0.253^***^	0.350^***^	-0.001	-2.177^***^	0.1428^***^	0.702^***^
	(0.0088)	(0.064)	(0.012)	(0.0475)	(0.0919)	(0.063)	(0.0047)	(0.4716)	(0.036)	(0.187)
${\Delta \tilde{i }}_{t-2}$	-0.0293^***^	0.4888^***^	-0.0126	0.738^***^	0.450^***^	0.910^***^	0.0124^**^	1.869^***^	-0.0730^**^	0.4879^***^
	(0.0100)	(0.068)	(0.014)	(0.0559)	(0.107)	(0.0917)	(0.0052)	(0.444)	(0.029)	(0.059)
${\Delta \tilde{i }}_{t-3}$	0.0016	0.018	0.0044	0.0926	0.251^**^	0.160^**^			-0.0245	-4.619^***^
	(0.0140)	(0.072)	(0.0167)	(0.057)	(0.110)	(0.0778)			(0.033)	(0.614)
${\Delta \tilde{i }}_{t-4}$					0.364^***^	0.285^***^				
					(0.111)	(0.079)				
$\theta$	0.0023	-0.129^***^	0.0088^*^	-0.377^***^	-0.175^***^	-0.295^***^	-0.001	0.366^*^	-0.026^***^	-0.1402^***^
	(0.002)	(0.018)	(0.0047)	(0.024)	(0.041)	(0.032)	(0.002)	(0.189)	(0.007)	(0.024)

* significant at 10% level, ** significant at 5% level, *** significant at 1% level. Standard errors in parentheses

**Table 17:** Smooth Transition Cointegrated Vector Autoregressive Model Results excluding Dummy Variables for Inflation Targeting Countries

	**CADNZD**		**CADSEK**		**AUDNZD**		**AUDSEK**		**NZDSEK**	
Independent Variables	Dependent Variables									
	${\Delta {\varvec{s}}}_{{\varvec{t}}}$	${\Delta \stackrel{\sim }{{\varvec{i}}}}_{{\varvec{t}}}$	${\Delta {\varvec{s}}}_{{\varvec{t}}}$	${\Delta \stackrel{\sim }{{\varvec{i}}}}_{{\varvec{t}}}$	${\Delta {\varvec{s}}}_{{\varvec{t}}}$	${\Delta \stackrel{\sim }{{\varvec{i}}}}_{{\varvec{t}}}$	${\Delta {\varvec{s}}}_{{\varvec{t}}}$	${\Delta \stackrel{\sim }{{\varvec{i}}}}_{{\varvec{t}}}$	${\Delta {\varvec{s}}}_{{\varvec{t}}}$	${\Delta \stackrel{\sim }{{\varvec{i}}}}_{{\varvec{t}}}$
**Regime 1**										
${\mu }_{0}$	0.0006^***^	-0.0003	0.00009	0.0012^***^	-0.1569^***^	-0.0336^***^	-0.0005	2.255^***^	-0.0004	1.0094^***^
	(0.0002)	(0.0003)	(0.0008)	(0.0004)	(0.0061)	(0.0017)	(0.0006)	(0.341)	(0.0007)	(0.0085)
${\Delta s}_{t-1}$	0.062^**^	0.0006	0.0025	0.0216	2.173^**^	-0.611	-0.0230^**^	4.938	-0.0078	0.3126
	(0.027)	(0.051)	(0.0135)	(0.0575)	(1.082)	(0.4367)	(0.0115)	(7.518)	(0.0129)	(1.4177)
${\Delta s}_{t-2}$	-0.0348	-0.034			0.549	-0.965^**^	0.0005	-1.063	-0.0141	-0.0641
	(0.0259)	(0.0503)			(1.120)	(0.421)	(0.0115)	(4.9488)	(0.0124)	(1.4175)
${\Delta s}_{t-3}$	0.0528^*^	-0.0213			-1.323	-0.870^**^	-0.0152	-1.997	-0.0098	0.3814
	(0.0286)	(0.0506)			(1.359)	(0.420)	(0.0113)	(8.491)	(0.0123)	(1.4197)
${\Delta \tilde{i }}_{t-1}$	0.0022	-0.2679^***^	-0.0112^***^	-0.0756^***^	-0.0071	-0.2076^***^	0.0021	1.616	-0.0017	0.5103^*^
	(0.0053)	(0.011)	(0.0034)	(0.0144)	(0.232)	(0.0756)	(0.0035)	(2.586)	(0.0027)	(0.2963)
${\Delta \tilde{i }}_{t-2}$	0.0017	-0.1125^***^			0.575^**^	-0.0102	0.0165^***^	1.373	0.0043	0.5276^*^
	(0.0053)	(0.0119)			(0.335)	(0.0759)	(0.0038)	(5.916)	(0.0029)	(0.3021)
${\Delta \tilde{i }}_{t-3}$	0.0052	-0.096^***^			0.6178^**^	-0.0710	-0.0003	9.881	0.0003	0.535^*^
	(0.0056)	(0.0116)			(0.289)	(0.072)	(0.0035)	(16.849)	(0.002)	(0.304)
$\theta$	-0.0030^**^	0.0021	-0.0011	-0.0201^***^	0.0682^***^	0.994^***^	-0.0026^***^	2.282	-0.0015^**^	-0.0144
	(0.0014)	(0.0026)	(0.0014)	(0.0078)	(0.011)	(0.011)	(0.0008)	(9.024)	(0.0008)	(0.0863)
**Regime 2**										
${\mu }_{0}$	-0.0017^***^	0.0145^***^	-0.0005^***^	-0.019^***^	0.015^**^	0.0388^***^	-0.0004	-1.292^***^	0.00009	0.953^**^
	(0.0004)	(0.002)	(0.0002)	(0.0039)	(0.0062)	(0.0049)	(0.0007)	(0.334)	(0.0001)	(0.379)
${\Delta s}_{t-1}$	-0.194^***^	0.095	-0.0105	-1.337^***^	-2.375^**^	-1.549	0.0918	-5.065	-0.0556^**^	-5.870
	(0.053)	(0.2445)	(0.0282)	(0.423)	(1.101)	(1.022)	(0.076)	(7.6296)	(0.028)	(4.557)
${\Delta s}_{t-2}$	0.070	-0.3658			-0.665	-0.381	0.9963^***^	0.750	0.1155^***^	3.111
	(0.063)	(0.288)			(1.138)	(1.1981)	(0.086)	(5.276)	(0.032)	(3.100)
${\Delta s}_{t-3}$	-0.1758^***^	0.1875			1.2147	-0.287	-1.180^***^	20.075	-0.056^*^	-1.994
	(0.0639)	(0.255)			(1.3788)	(1.237)	(0.172)	(86.029)	(0.0334)	(18.477)
${\Delta \tilde{i }}_{t-1}$	-0.013	-0.864^***^	0.012^*^	0.133^**^	-0.154	-0.5059^**^	-0.063	-0.891	0.0115^**^	4.1209
	(0.013)	(0.063)	(0.006)	(0.061)	(0.236)	(0.1967)	(0.0472)	(2.736)	(0.0056)	(9.343)
${\Delta \tilde{i }}_{t-2}$	-0.001	-0.128^**^			-0.718^**^	-0.666^***^	-8.083^***^	-13.243	0.0024	7.530^*^
	(0.013)	(0.055)			(0.338)	(0.245)	(1.658)	(59.959)	(0.0052)	(4.366)
${\Delta \tilde{i }}_{t-3}$	-0.024^*^	-0.090^*^			-0.7247^**^	-0.721^***^	-0.010	-9.944	-0.0162^*^	0.571
	(0.013)	(0.051)			(0.291)	(0.266)	(0.251)	(16.867)	(0.0093)	(2.517)
$\theta$	0.0052	0.079^***^	-0.012^***^	0.500^***^	0.0328^***^	0.0347	0.0158^*^	-2.334	-0.0019	1.087
	(0.0036)	(0.019)	(0.0029)	(0.085)	(0.011)	(0.0285)	(0.009)	(9.171)	(0.002)	(3.564)

* significant at 10% level, ** significant at 5% level, *** significant at 1% level. Standard errors in parentheses

**Table 18:** Smooth Transition Cointegrated Vector Autoregressive Model Results excluding Dummy Variables for Non-Targeting Economies

	**USDEUR**		**USDCHF**		**EURCHF**	
Independent Variables	Dependent Variables					
	${\Delta {\varvec{s}}}_{{\varvec{t}}}$	${\Delta \stackrel{\sim }{{\varvec{i}}}}_{{\varvec{t}}}$	${\Delta {\varvec{s}}}_{{\varvec{t}}}$	${\Delta \stackrel{\sim }{{\varvec{i}}}}_{{\varvec{t}}}$	${\Delta {\varvec{s}}}_{{\varvec{t}}}$	${\Delta \stackrel{\sim }{{\varvec{i}}}}_{{\varvec{t}}}$
**Regime 1**						
${\mu }_{0}$	0.0004^*^	0.00005	0.0005	-0.0017	-0.00005	0.0015
	(0.0002)	(0.0002)	(0.0007)	(0.0033)	(0.00005)	(0.0019)
${\Delta s}_{t-1}$	0.1406^***^	0.0092	-0.016	-0.102	-0.0132	0.6440
	(0.0337)	(0.046)	(0.014)	(0.684)	(0.0246)	(0.6724)
${\Delta s}_{t-2}$	-0.084^**^	0.0596	0.013	-0.2418		
	(0.039)	(0.0461)	(0.0126)	(0.543)		
${\Delta \tilde{i }}_{t-1}$	-0.0083	0.055^***^	0.0001	0.0289	-0.0003	-0.248^***^
	(0.0069)	(0.0112)	(0.0005)	(0.5300)	(0.0003)	(0.0114)
${\Delta \tilde{i }}_{t-2}$	-0.0113^*^	-0.0996^***^	-0.0002	0.0218		
	(0.0063)	(0.011)	(0.0005)	(0.2945)		
$\theta$	-0.0005	-0.0004	-0.0008^**^	-0.0334	0.0002	-0.076^***^
	(0.0003)	(0.0004)	(0.0004)	(0.0345)	(0.0002)	(0.005)
**Regime 2**						
${\mu }_{0}$	-0.0004	-0.197^***^	0.0006^**^	0.0011	0.0004^***^	-0.051^***^
	(0.0002)	(0.013)	(0.0003)	(0.004)	(0.0001)	(0.010)
${\Delta s}_{t-1}$	-0.153^***^	-23.106^***^	0.173^***^	0.255	0.270^***^	-0.232
	(0.0358)	(1.655)	(0.0339)	(0.7954)	(0.034)	(1.1838)
${\Delta s}_{t-2}$	0.0965^**^	0.902	-0.0459	0.288		
	(0.041)	(1.738)	(0.0418)	(0.738)		
${\Delta \tilde{i }}_{t-1}$	0.0085	-1.143^***^	0.0015	-0.521	0.0009	-0.2217^***^
	(0.007)	(0.067)	(0.001)	(0.530)	(0.0006)	(0.0427)
${\Delta \tilde{i }}_{t-2}$	0.0117^*^	1.072^***^	0.0012	-0.2852		
	(0.0069)	(0.064)	(0.0011)	(0.2948)		
$\theta$	0.0005	0.227^***^	0.0029	0.0338	-0.0008^**^	-0.1576^***^
	(0.0003)	(0.026)	(0.0012)	(0.0347)	(0.0004)	(0.027)
* significant at 10% level, ** significant at 5% level, *** significant at 1% level. Standard errors in parentheses						

It is also important to provide evidence that the chosen transition variables are in fact suitable. Table 19 compares the adjustment speed of the original model, which includes the change in the 30-day interest rate as a transition variable, with those from models with alternative transition variables. As can be seen, the highest speed is estimated in the case of the specification including the 30-day interest rate as the transition variable.

**Table 19:** Adjustment Speed in Smooth Transition CVAR Model With Different Transition Variables

		**Change in the 30-day interest rate**		**Level of the relative 30-day**		**Absolute change in the 30-day interest rate**		**30-day interest rate minus the overnight rate**		**10-year interest rate minus the overnight rate**	
		Regime 1	Regime 2	Regime 1	Regime 2	Regime 1	Regime 2	Regime 1	Regime 2	Regime 1	Regime 2
GBPCAD	$\Delta {x}_{t}$	0.0006^**^	0.0006	-0.0037	0.0037	0.0001	-0.0008	-0.0005	-0.0027	-0.005^***^	0.0057^***^
	$\Delta {\tilde{i }}_{t}$	0.069^***^	-0.133^***^	-0.0036	-0.025^***^	-0.0024	-0.055^***^	-0.15^***^	0.139^***^	-0.168^***^	0.1566^***^
GBPAUD	$\Delta {x}_{t}$	-0.002^***^	0.005	0.023	-0.0245	-0.002^***^	0.0067	0.0014	-0.0032	-0.0014	0.0004
	$\Delta {\tilde{i }}_{t}$	0.203^***^	-0.379^***^	0.0129	-0.028^***^	-0.0015	-0.033^***^	-0.177^***^	0.180^***^	-0.214^***^	0.2364^***^
GBPNZD	$\Delta {x}_{t}$	0.114^***^	-0.174^***^	0.013^***^	-0.152^***^	-0.1047	0.1048	0.0998^***^	0.0013	0.0102^***^	-0.080^***^
	$\Delta {\tilde{i }}_{t}$	0.169^***^	-0.298^***^	-0.009^***^	0.0088	0.0046	-0.107^***^	0.0601^***^	-0.069^***^	-0.030^***^	0.0380^***^
GBPSEK	$\Delta {x}_{t}$	-0.0006	-0.0013	0.0016	-0.0026	-0.0009	-0.0002	-0.0029	0.0021	0.0021^*^	-0.005^***^
	$\Delta {\tilde{i }}_{t}$	0.319^***^	-0.323^***^	-0.083^***^	0.553^***^	-0.0347	0.047^*^	0.0493^***^	-0.089^***^	0.0787^***^	-0.132^***^
CADAUD	$\Delta {x}_{t}$	0.009^**^	-0.02^***^	-0.0167	0.0137	-0.0015	-0.0104^*^	-0.010^**^	0.0074	-0.0041^*^	0.0019
	$\Delta {\tilde{i }}_{t}$	0.201^***^	-0.200^***^	0.0026	-0.0005	0.0002	0.059^***^	0.1123^***^	-0.11^***^	0.0345^**^	-0.0322^**^
CADNZD	$\Delta {x}_{t}$	-0.002^**^	0.004	-0.235^*^	0.2338^*^	-0.0016^**^	0.0025	-0.0006	-0.0124	0.0034	-0.0048
	$\Delta {\tilde{i }}_{t}$	0.0021	0.100^***^	0.0021	0.0574^*^	0.0008	0.1041^***^	0.1554^***^	-0.155^***^	0.0458^**^	-0.0458^**^
CADSEK	$\Delta {x}_{t}$	-0.001	-0.011^***^	-0.0026^*^	-0.0016	0.0015	-0.009^***^	-0.004^***^	-0.0009	-0.004^***^	0.0014
	$\Delta {\tilde{i }}_{t}$	-0.016^**^	-0.425^***^	-0.0054	0.0252^***^	-0.0067	0.3645^***^	0.0126^*^	-0.0121	-0.0063	0.0158
AUDNZD	$\Delta {x}_{t}$	0.067	0.034	-0.085^***^	-0.270^***^	0.0106^***^	-0.242^**^	0.009^***^	0.0116^***^	0.0885^***^	0.1525^***^
	$\Delta {\tilde{i }}_{t}$	0.100^***^	-0.330^***^	-0.046^***^	-0.0048	0.0103^***^	-0.0584	0.0591^***^	-0.071^***^	0.0471^***^	-0.060^***^
AUDSEK	$\Delta {x}_{t}$	-0.001	-0.016^**^	-0.0672	-0.056	-0.003^***^	0.0214^**^	0.0002	-0.0029	-0.038^**^	0.0354^**^
	$\Delta {\tilde{i }}_{t}$	0.136	-0.378^***^	-0.286	0.0259	0.095^**^	-10.586^**^	0.0805^*^	0.078^***^	0.0522	-0.0191
NZDSEK	$\Delta {x}_{t}$	-0.0015	-0.002	-0.0019^**^	0.0004	-0.002^***^	0.0104^***^	-0.0020^*^	0.0089^**^	-0.002^***^	-0.036^***^
	$\Delta {\tilde{i }}_{t}$	0.006	-0.383^***^	0.0476^***^	-0.057^***^	0.0006	-0.0739	-0.154^***^	0.0345^***^	-0.0209	0.0202^***^
USDEUR	$\Delta {x}_{t}$	-0.0003	0.0004	-0.0007	-0.0002	-0.0004	-0.001	0.0007	-0.0003	0.0007	-0.006^***^
	$\Delta {\tilde{i }}_{t}$	-0.0004	-0.209^***^	-0.0036^**^	0.0031^**^	-0.0003	-0.078^***^	-0.0009	-0.001	-0.0004	-0.017^***^
USDCHF	$\Delta {x}_{t}$	-0.0009	0.0009	0.0055^**^	-0.0063^**^	-0.002^***^	0.004^***^	-0.0008	0.0002	-0.041^***^	0.040^***^
	$\Delta {\tilde{i }}_{t}$	-0.032^***^	-0.132^***^	-0.062^***^	0.0329^*^	0.0074	-0.0273^**^	-0.081^***^	0.0807^***^	-0.104^***^	0.104^***^
EURCHF	$\Delta {x}_{t}$	0.00014	-0.0008^**^	-0.003^***^	0.0033^***^	0.0003	-0.001^***^	-0.0004^**^	0.0007^*^	-0.0002^*^	0.0006
	$\Delta {\tilde{i }}_{t}$	-0.076^***^	-0.182^***^	-0.0193^*^	-0.094^***^	-0.025^***^	-0.132^***^	-0.083^***^	0.0309	-0.112^***^	-0.403^***^
* significant at 10% level, ** significant at 5% level, *** significant at 1% level. $\Delta {x}_{t}$ indicates the exchange rate equation $\Delta {\tilde{i }}_{t}$ indicates the interest rate differential equation											

## Conclusions

This paper tests for the existence of a UIP-type relationship between the exchange rate and the interest differential by estimating first a benchmark linear Cointegrated VAR including the nominal exchange rate and the interest rate differential as well as central bank announcements, and then a Smooth Transition Cointegrated VAR (STCVAR) model incorporating nonlinearities and also taking into account the role of interest rate expectations. The analysis is conducted for five inflation targeting countries (the UK, Canada, Australia, New Zealand and Sweden) and also, for comparison purposes, for three non-targeters (the US, the Euro-Area and Switzerland) using daily data from January 2000 to December 2020.

While we cannot find a UIP relationship in its strictest sense, we provide plenty of evidence of a long-run equilibrium relationship between the exchange rate and the interest rate differential. The main findings can be summarised as follows. First, the nonlinear framework appears to be more appropriate than the linear one to capture the adjustment towards the long-run equilibrium relationship between the exchange rate and the interest rate differential, which is consistent with the results of other related studies (see, for example, Sarno et al. 2005, 2006; Li et al. 2013). The estimated speed of adjustment is substantially faster in the nonlinear model, which lends greater support to the existence of a long run equilibrium than the linear one; similarly, the short-run dynamic linkages appear to be more significant in the nonlinear case. Second, interest rate expectations, a measure of central bank credibility which is often neglected in the context of investigating the long-run equilibrium relationship between the exchange rate and the interest rate differential, play an important role. In particular, a fast adjustment only occurs when the market expects the interest rate to increase in the near future, which suggests that central banks are perceived as more credible when sticking to their goal of keeping inflation at a low and stable rate. The change in the 30-day interest rate appears to be the most appropriate of the transition variables considered as indicated by the corresponding adjustment speeds. Third, central bank announcements have a more sizeable short- run effect in the nonlinear model which also includes interest rate expectations. Fourth, the long-run equilibrium holds better in inflation targeting countries, where the adjustment speed is faster than in non-targeting economies. This suggests that, in general, the inflation targeting framework tends to generate a higher degree of credibility for monetary authorities, thereby reducing deviations of the exchange rate from the long-run equilibrium.

## Figures and Tables

**Fig. 1:**
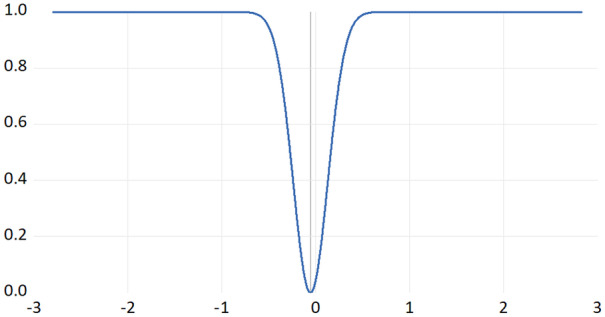
GBPCAD – Exchange Rate Equation

**Fig. 2:**
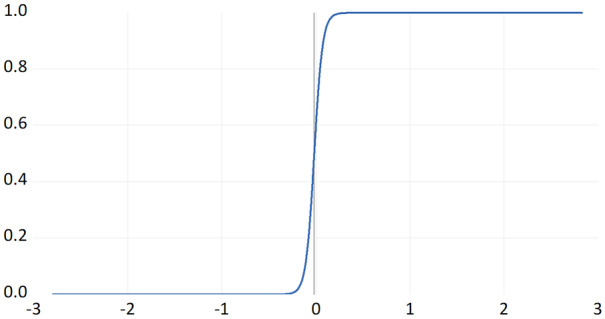
GBPCAD – Interest Rate Equation

**Fig. 3:**
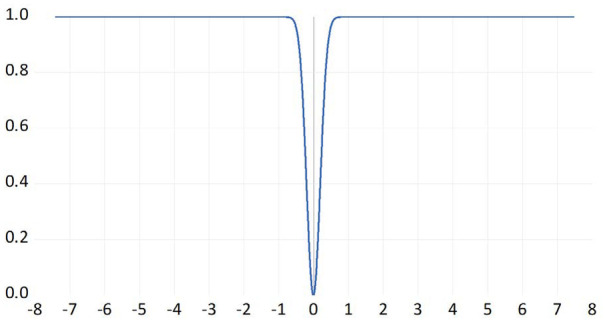
GBPAUD – Exchange Rate Equation

**Fig. 4:**
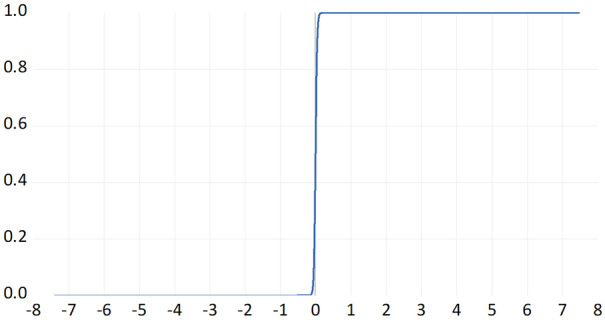
GBPAUD – Interest Rate Equation

**Fig. 5:**
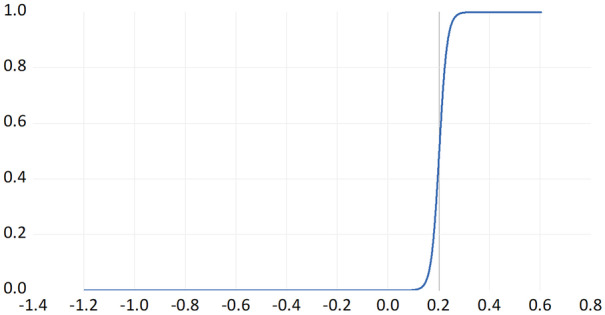
GBPNZD – Exchange Rate Equation

**Fig. 6:**
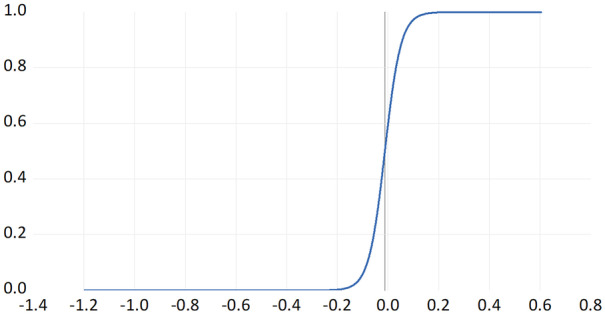
GBPNZD – Interest Rate Equation

**Fig. 7:**
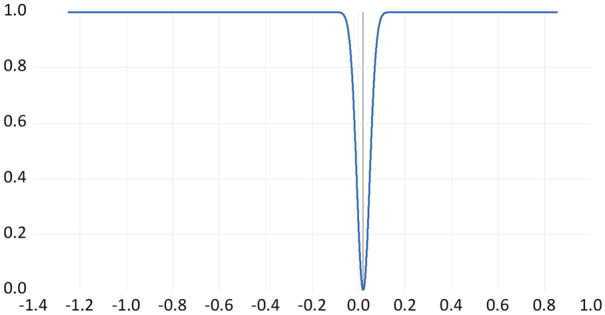
GBPSEK – Exchange Rate Equation

**Fig. 8:**
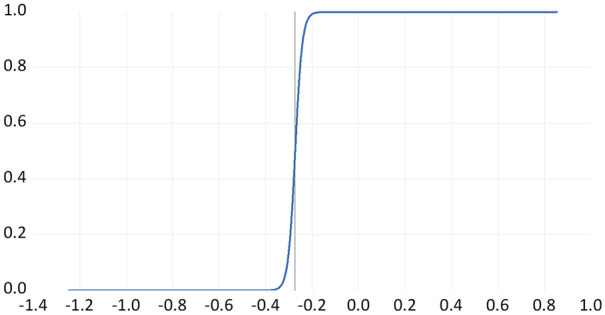
GBPSEK – Interest Rate Equation

**Fig. 9:**
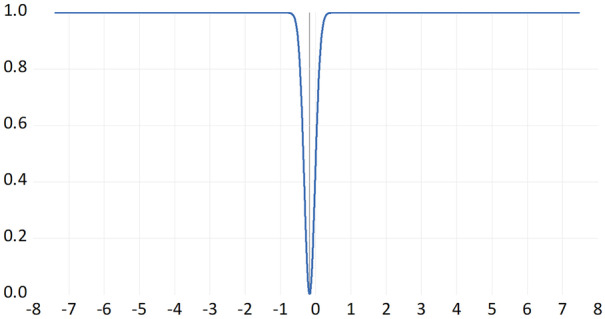
CADAUD – Exchange Rate Equation

**Fig. 10:**
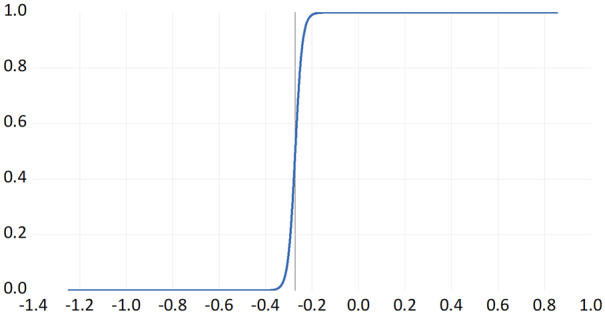
CADAUD – Interest Rate Equation

**Fig. 11:**
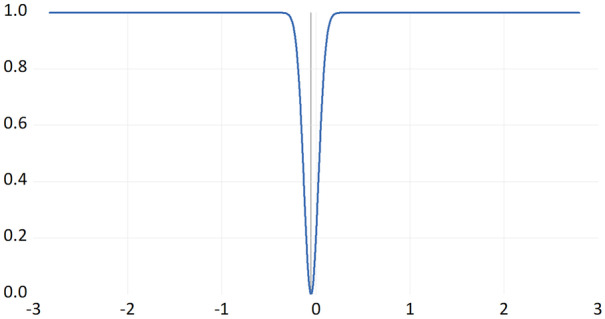
CADNZD – Exchange Rate Equation

**Fig. 12:**
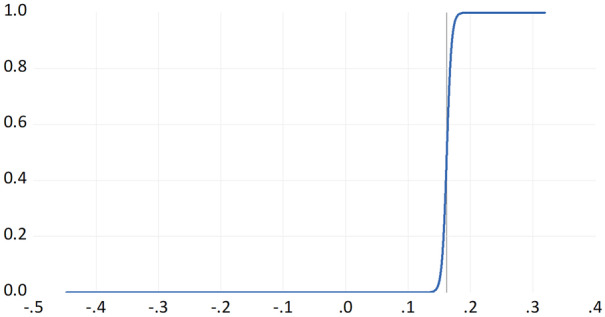
CADNZD – Interest Rate Equation

**Fig. 13:**
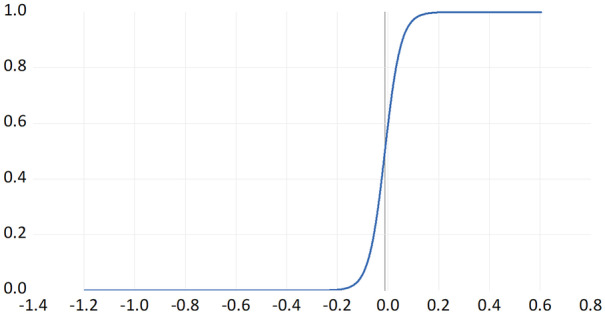
CADSEK – Exchange Rate Equation

**Fig. 14:**
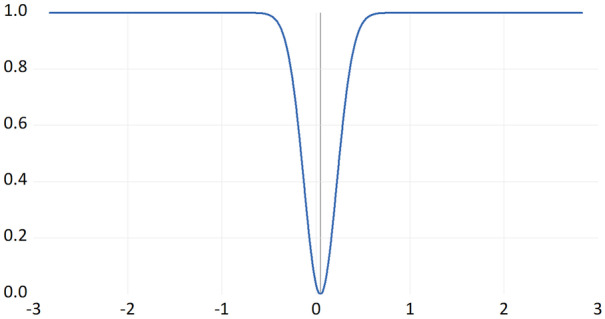
CADSEK – Interest Rate Equation

**Fig. 15:**
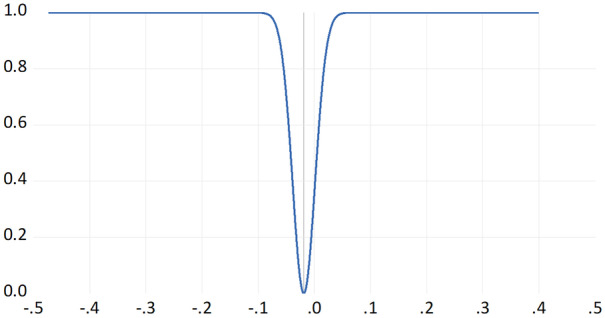
AUDNZD – Exchange Rate Equation

**Fig. 16:**
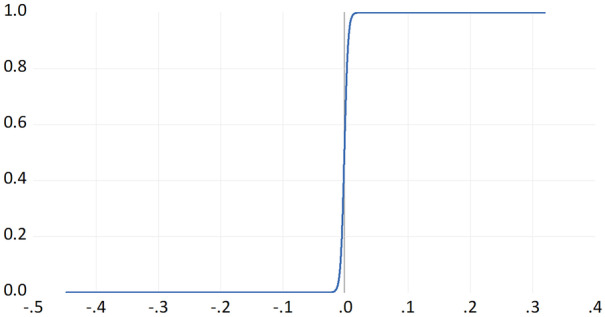
AUDNZD – Interest Rate Equation

**Fig. 17:**
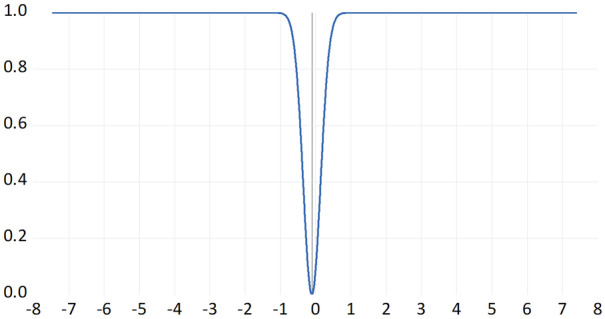
AUDSEK – Exchange Rate Equation

**Fig. 18:**
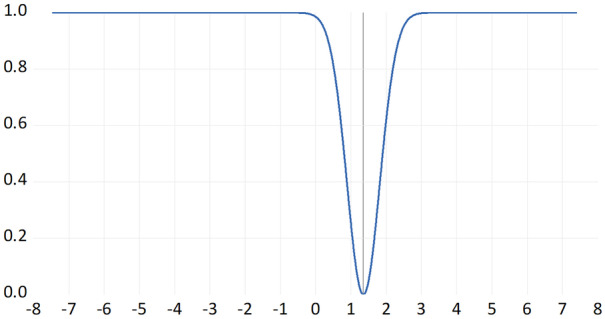
AUDSEK – Interest Rate Equation

**Fig. 19:**
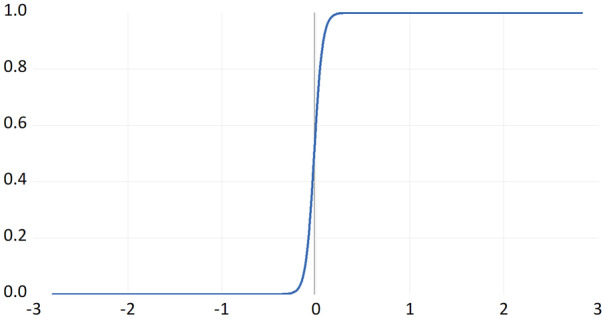
NZDSEK – Exchange Rate Equation

**Fig. 20:**
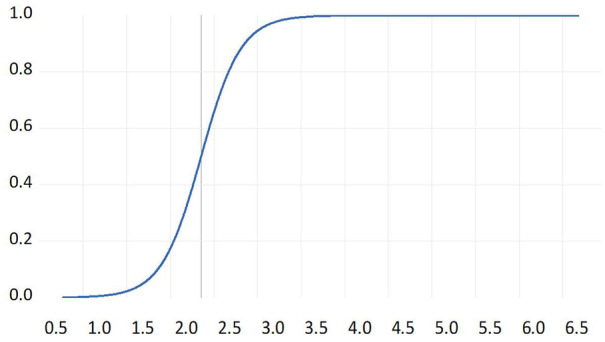
NZDSEK – Interest Rate Equation

**Fig. 21:**
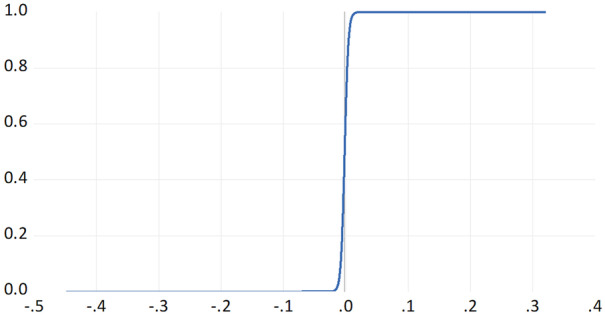
USDEUR – Exchange Rate Equation

**Fig. 22:**
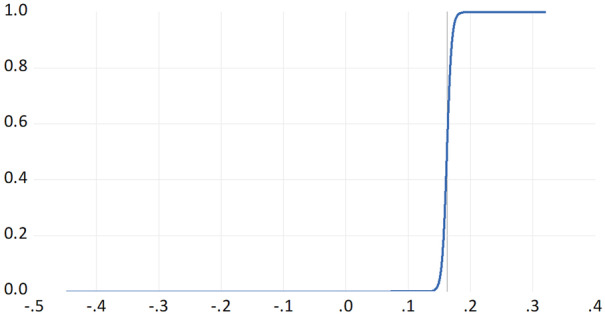
USDEUR – Interest Rate Equation

**Fig. 23:**
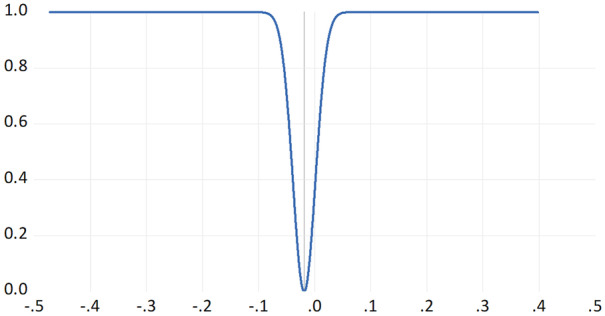
USDCHF – Exchange Rate Equation

**Fig. 24:**
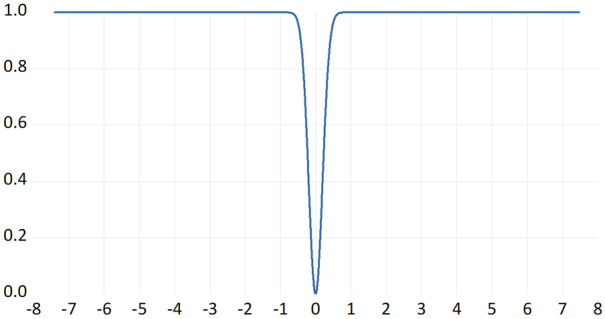
USDCHF – Interest Rate Equation

**Fig. 25:**
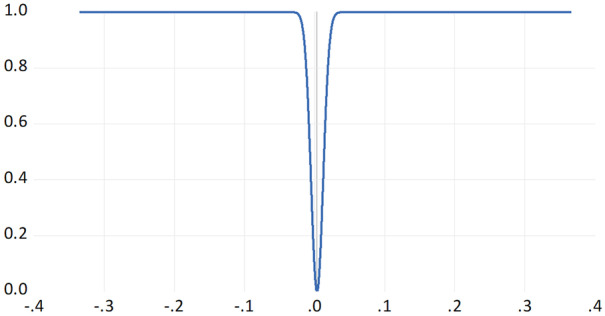
EURCHF – Exchange Rate Equation

**Fig. 26:**
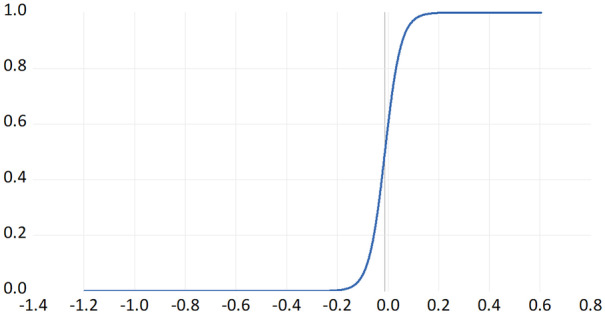
EURCHF – Interest Rate Equation

## Footnotes

1 We also estimated a trivariate model with the level of the exchange rate, the domestic and the foreign interest rate, and tested the restriction that the two latter variables have equal but opposite coefficients. A LR test confirms that this is a valid restriction and that therefore the bivariate model is appropriate. These results are not reported.
